# Misregulation of Alternative Splicing in a Mouse Model of Rett Syndrome

**DOI:** 10.1371/journal.pgen.1006129

**Published:** 2016-06-28

**Authors:** Ronghui Li, Qiping Dong, Xinni Yuan, Xin Zeng, Yu Gao, Cassandra Chiao, Hongda Li, Xinyu Zhao, Sunduz Keles, Zefeng Wang, Qiang Chang

**Affiliations:** 1 CMB Training Program, University of Wisconsin-Madison, Madison, Wisconsin, United States of America; 2 Waisman Center, University of Wisconsin-Madison, Madison, Wisconsin, United States of America; 3 Department of Pharmacology, University of North Carolina at Chapel Hill, Chapel Hill, North Carolina, United States of America; 4 Department of Statistics, University of Wisconsin-Madison, Madison, Wisconsin, United States of America; 5 Genetics Training Program, University of Wisconsin-Madison, Madison, Wisconsin, United States of America; 6 Department of Biostatistics and Medical Informatics, University of Wisconsin-Madison, Madison, Wisconsin, United States of America; 7 Chinese Academy of Sciences (CAS) Key Laboratory of Computational Biology, CAS-MPG Partner Institute for Computational Biology, Shanghai, China; 8 Departments of Medical Genetics and Neurology, University of Wisconsin-Madison, Madison, Wisconsin, United States of America; National Cancer Institute, UNITED STATES

## Abstract

Mutations in the human *MECP2* gene cause Rett syndrome (RTT), a severe neurodevelopmental disorder that predominantly affects girls. Despite decades of work, the molecular function of MeCP2 is not fully understood. Here we report a systematic identification of MeCP2-interacting proteins in the mouse brain. In addition to transcription regulators, we found that MeCP2 physically interacts with several modulators of RNA splicing, including LEDGF and DHX9. These interactions are disrupted by RTT causing mutations, suggesting that they may play a role in RTT pathogenesis. Consistent with the idea, deep RNA sequencing revealed misregulation of hundreds of splicing events in the cortex of *Mecp2* knockout mice. To reveal the functional consequence of altered RNA splicing due to the loss of MeCP2, we focused on the regulation of the splicing of the flip/flop exon of *Gria2* and other AMPAR genes. We found a significant splicing shift in the flip/flop exon toward the flop inclusion, leading to a faster decay in the AMPAR gated current and altered synaptic transmission. In summary, our study identified direct physical interaction between MeCP2 and splicing factors, a novel MeCP2 target gene, and established functional connection between a specific RNA splicing change and synaptic phenotypes in RTT mice. These results not only help our understanding of the molecular function of MeCP2, but also reveal potential drug targets for future therapies.

## Author Summary

Rett syndrome (RTT) is a debilitating neurodevelopmental disorder with no cure or effective treatment. To fully understand the disease mechanism and develop therapies, it is necessary to study all aspects of the molecular function of methyl-CpG binding protein 2 (MeCP2), mutations in which have been identified as the genetic cause of RTT. Over the years, MeCP2 has been shown to maintain DNA methylation, regulate transcription and chromatin structure, control microRNA processing, and modulate RNA splicing. Among these known functions, the role of MeCP2 in modulating RNA splicing is less well understood. We took several unbiased approaches to investigate the how MeCP2 may regulate splicing, what splicing changes are caused by the loss of MeCP2, and what functional consequences are caused by altered splicing. We discovered that MeCP2 interacts with splicing factors to regulated the splicing of glutamate receptor genes, which mediate the vast majority of excitatory synaptic transmission in the brain; and linked the altered splicing of glutamate receptor genes to specific synaptic changes in a RTT mouse model. Our findings not only advance the understanding of RTT disease mechanism, but also reveal a potential drug target for future development of therapies.

## Introduction

Rett syndrome (RTT) is a progressive neurodevelopmental disorder that predominantly affects females[1, 2]. Classic RTT patients develop normally in the first 6–18 months, and then undergo a rapid regression of higher brain functions that eventually leads to the loss of speech and purposeful hand movement, microcephaly, dementia, ataxia and seizure[3]. Mutations in the human X-linked methyl-CpG-binding protein 2 (*MECP2*) gene are responsible for over 90% of classic RTT cases[4]. MeCP2 is abundantly expressed in the mammalian central nervous system (CNS) and binds to methylated CpG site throughout the genome[5]. Despite decades of work, the underlying molecular mechanism of how mutations of *MECP2* lead to RTT is not fully understood.

In order to reveal the RTT disease mechanism, it is necessary to study the molecular function of MeCP2. Previous research on the molecular function of MeCP2 has focused on the localization of MeCP2 in the nucleus and the proteins that physically interact with MeCP2. At the microscopic level, MeCP2 appears to be colocalized with heterochromatin and thus is hypothesized to induce large-scale chromatin reorganization during terminal differentiation[6]. At the genomic level, MeCP2 can bind to unmethylated DNA[7], methylated cytosine[5], and hydroxymethylated cytosine[8], and may preferentially modulate the expression of long genes[9]. In parallel to research on MeCP2 localization, many proteins have been identified to physically interact with MeCP2. Based on the known functions of identified MeCP2-interacting proteins, previous studies have suggested a role for MeCP2 in maintaining DNA methylation[10], regulating transcription[11–16], chromatin structure[17–22], and RNA processing[23–25]. Future effort to combine the insights from the two approaches described above may allow more detailed understanding of the regulation of each of these specific protein-protein interactions across the entire genome, as well as the relevance of each interaction to RTT disease pathogenesis.

Misregulation of RNA alternative splicing has been implicated in a number of neurological disorders, which can be classified into two categories: cis-acting splicing disorder and trans-acting disorder[26]. Cis-acting disorder is caused by mutations that affect splicing of the mutant gene itself and therefore the function of that gene. An example of this type is frontotemporal dementia with Parkinsonism linked to chromosome 17 (FTDP-17), in which mutations in the *MAPT* (Tau) gene alter the function of Tau by increasing the exon 10 containing isoform[27]. In contrast, trans-acting disorders are caused by the loss of function of genes with regulatory roles in RNA splicing. For instance, the loss of survival motor neuron protein 1 (SMN1) function affects biogenesis of small nuclear RNA (snRNA) and lead to widespread splicing changes in spinal muscular atrophy (SMA)[28]. Relevant to RTT, the Zoghbi lab has identified RNA-dependent interaction between MeCP2 and Y box-binding protein 1 (YB1) in cultured cells, and further reported many altered RNA splicing events in a mouse model of RTT[23]. However, it is not clear if the splicing alterations are indeed dependent on the MeCP2/YB1 interaction and no link has been discovered between any gene-specific splicing change and specific neuronal phenotypes in RTT. Therefore the mechanistic and functional links between MeCP2, splicing regulation, and RTT phenotypes remain elusive.

To facilitate systematic identification of MeCP2-interacting proteins in the brain, we created a knockin mouse line (*Mecp2-Flag*) that expresses Flag-tagged MeCP2 from the endogenous *Mecp2* locus[29]. This unique tool gives us two main advantages. First, it ensures that the MeCP2-Flag protein is expressed at the physiological level, so that non-specific protein-protein interactions caused by the overexpression of MeCP2-Flag is minimized. Second, it allows us to use a highly efficient anti-Flag antibody in the co-immunoprecipitation. The choice of antibody is not a trivial issue, because in the past, different anti-MeCP2 antibodies in co-immunoprecipitation experiments have generated conflicting results in the identification of MeCP2-interacting proteins[30, 31]. Mass spectrometry analysis of proteins co-immunoprecipitated by the anti-Flag antibody from the nuclear extract prepared from the *Mecp2-Flag* mouse brains showed that MeCP2 interacted with multiple splicing factors. Some of these physical interactions were disrupted by RTT-causing mutations in MeCP2. Furthermore, ChIP-seq analysis of revealed MeCP2 occupancy at exon/intron injections, which provides additional support of the role for MeCP2 in modulating alternative splicing. Consistent with previous findings, hundreds of splicing events were found to be misregulated in the cortex of *Mecp2* knockout (KO) mice. More importantly, a specific splicing change in the *Mecp2* KO cortex-a shift in the balance between the flip and flop exon in the AMPA receptor (AMPAR) genes was causally linked to synaptic phenotypes of faster desensitization kinetics of AMPAR-gated current and altered synaptic transmission. Together, our findings substantiate the role of MeCP2 in regulating alternative splicing of RNA by revealing direct physical interaction between MeCP2 and multiple splicing factors, association of MeCP2 at exon/intron junction, and providing the first functional link between a specific splicing alteration and synaptic phenotypes in RTT mice.

## Results

### Systematic identification of MeCP2-interacting proteins in the brain

**Fig 1:**
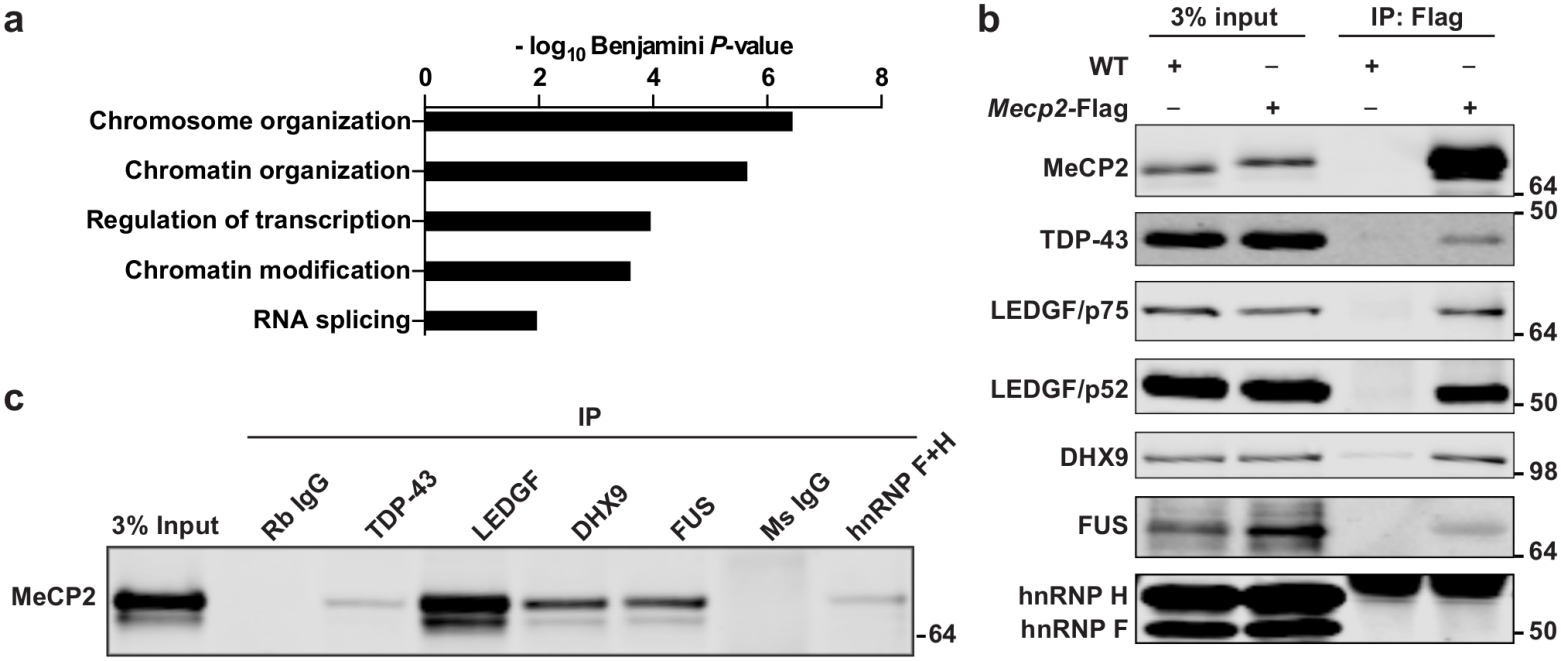
MeCP2 interacts with multiple splicing regulators. (a) Gene ontology (GO) analysis of proteins identified from anti-Flag co-immunoprecipitation and mass spectrometry (Co-IP/MS) analysis in *Mecp2-Flag* knockin mouse brain. Top five GOTERM_BP_FAT terms were shown and –log_10_ Benjamini *P*-value is plotted. (b) Western blot validation of interactions between MeCP2 and splicing regulators identified in Co-IP/MS. Anti-Flag immunoprecipitate in nuclear extract from WT and *Mecp2-Flag* knockin mouse brain is resolved in SDS-PAGE and probed with antibodies against indicated proteins. (c) Western blot analysis of MeCP2 in anti-TDP-43, anti-LEDGF, anti-DHX9, anti-FUS and anti-hnRNP F+H immunoprecipitate. Rabbit (Rb) IgG is negative control for rabbit antibody (anti-TDP-43, LEDGF, DHX9 and FUS) and mouse (Ms) IgG is negative control for mouse antibody (anti-hnRNP F+H).

To facilitate identification of MeCP2-interacting proteins in the mouse brain, we generated the *Mecp2-Flag* knockin mouse line that expresses Flag-tagged wild-type MeCP2 from the endogenous locus. We purified nuclei from the brains of male *Mecp2-Flag* mice, prepared nuclear extract and performed co-immunoprecipitation (co-IP) using the anti-Flag antibody. Eluted protein sample was then subjected to protein identification by mass spectrometry. Forty-eight proteins were identified using highly stringent statistical filters (S1 Table). Identified proteins included previously known MeCP2-interacting transcriptional regulators and chromatin remodeling proteins, such as HDAC1 and components of the SWI/SNF complex. Consistently, gene ontology (GO) analysis showed that proteins identified by co-IP/MS were enriched with GO terms of chromatin organization, chromatin modification and regulation of transcription. Interestingly, these proteins were also enriched for RNA splicing (Fig 1a). MeCP2 has been implicated in regulating splicing, but its role in pre-mRNA splicing has not been studied extensively. Therefore we decided to focus our study on the interaction between MeCP2 and several splicing factors.

To validate the physical interaction between MeCP2 and splicing factors, we performed anti-Flag co-IP in nuclear extract from the *Mecp2-Flag* knockin mouse brain and probed it with antibodies against TDP-43, LEDGF, DHX9, FUS, hnRNP H, and hnRNP F, respectively. Western blot results showed that TDP-43, LEDGF, DHX9 and FUS were co-immunoprecipitated with MeCP2, whereas hnRNP H and hnRNP F were not (Fig 1b). Next, we performed reverse co-IP and detected MeCP2 in immunoprecipitate of anti-TDP-43, anti-LEDGF, anti-DHX9, anti-FUS, and anti-hnRNPH+F (Fig 1c), further confirming that MeCP2 physically interacts with these proteins in the mouse brain. In addition, the interaction between MeCP2 and splicing factors were not sensitive to Benzonase treatment, which digest and remove all nucleic acids, suggesting that these interactions were most likely direct interactions independent of either DNA or RNA (S1 Fig).

### RTT mutations disrupt interactions between MeCP2 and LEDGF or DHX9

**Fig 2:**
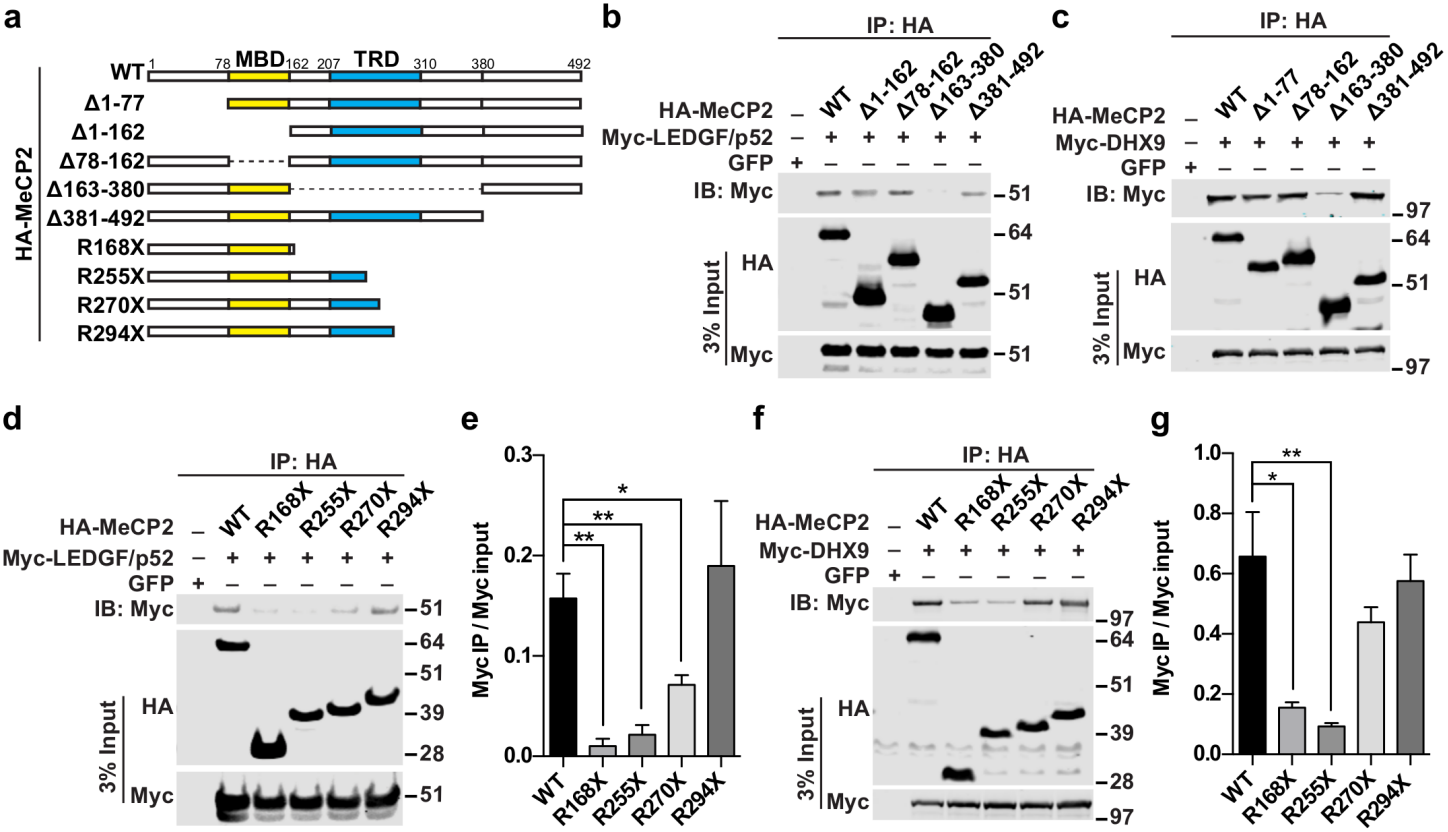
RTT mutations disrupt MeCP2 interaction with LEDGF and DHX9. (a) Schematic diagram of HA-MeCP2 constructs used in this study. The methyl-CpG-binding domain (MBD) domain is shown in yellow and the transcription repression domain (TRD) in blue. (b) Western blot analysis of Myc-LEDGF/p52 in anti-HA immunoprecipitate (top panel) from cells transfected with different combinations of plasmids as labeled on top. HA-MeCP2 (middle panel) and Myc-LEDGF/p52 (bottom panel) in input were also analyzed by western blot. (c) Western blot analysis of Myc-DHX9 in anti-HA immunoprecipitate (top panel) from cells transfected with different combinations of plasmids as labeled on top. HA-MeCP2 (middle panel) and Myc-DHX9 (bottom panel) in input were also analyzed by Western blot. (d) Representative western blot analysis of Myc-LEDGF/p52 in HA immunoprecipitate (top panel) from cells transfected with different combinations of plasmids as labeled on top. HA-MeCP2 (middle panel) and Myc-LEDGF/p52 (bottom panel) in input were also analyzed by Western blot. (e) Quantification of Myc-LEDGF/p52 IP over input signal intensity of three independent experiments. Error bar represents S.E.M, **P < 0.01, *P < 0.05; two-tailed *t*-test. (f) Representative Western blot analysis of Myc-DHX9 in HA immunoprecipitate (top panel) from cells transfected with different combinations of plasmids as labeled on top. HA-MeCP2 (middle panel) and Myc-DHX9 (bottom panel) in input were also analyzed by Western blot. (g) Quantification of Myc-DHX9 IP over input signal intensity of four independent experiments. Error bar represents S.E.M, **P < 0.01, *P < 0.05; two-tailed *t*-test.

The interaction between MeCP2 and LEDGF has been previously reported in cancer cells[32], but not in the brain. MeCP2 interacts with the N-terminal PWWP-CR1 domain of LEDGF, but which domain of MeCP2 that LEDGF binds to is not defined. DHX9 has recently been revealed as an interacting partner of MeCP2[33], but the interaction domain is not known either. To examine which domain of MeCP2 is required for interaction with LEDGF and DHX9, we expressed HA-tagged MeCP2 with different deletions (Fig 2a) and Myc-tagged full-length LEDGF/p52, LEDGF/p75 and DHX9, respectively, in HEK293 cells. Co-IP with anti-HA antibody followed by Western blot with anti-Myc antibody showed that deletion of amino acids 163–380 of the MeCP2 protein significantly reduced the interaction between MeCP2 and LEDGF/p52, LEDGF/p75 or DHX9 (Fig 2b and 2c; S2a Fig). Reverse co-IP with anti-Myc antibody followed by Western blot with anti-HA antibody also demonstrated that amino acids 163–380 of the MeCP2 protein was required for interaction between MeCP2 and LEDGF/p52 (S2b Fig). Collectively, these results strongly suggested that the transcription repression domain (TRD) of MeCP2 is essential for the interaction of MeCP2 with RNA binding proteins.

Several RTT disease causing mutations locate in the region of amino acids 163–380 (R168X, R255X, R270X and R294X) and may disrupt the TRD domain, therefore we asked whether these mutations affect the interaction between MeCP2 and LEDGF or DHX9. To test this, we co-transfected MeCP2 constructs encoding MeCP2 WT, MeCP2^R168X^, MeCP2^R255X^, MeCP2^R270X^, and MeCP2^R294X^, respectively, with LEDGF/p52 or DHX9 in HEK293 cells. Co-IP assay showed that interaction between LEDGF/p52 and MeCP2^R168X^, MeCP2^R255X^, and MeCP2^R270X^ was significantly impaired (Fig 2d and 2e). Interestingly, the interaction between MeCP2^R294X^ (retaining a large fraction of TRD) and LEDGF/p52 was not significantly different from that between wild type MeCP2 and LEDGF/p52, suggesting that amino acids 270–294 of MeCP2 are required for its binding to LEDGF/p52 (Fig 2d and 2e). Similarly, we found that MeCP2^R168X^ and MeCP2^R255X^ interacted poorly with DHX9, while MeCP2^R270X^ and MeCP2^R294X^ had intact binding capability, indicating that amino acids 255–270 of MeCP2 are required for its binding to DHX9 (Fig 2f and 2g).

### Misregulation of alternative splicing in the cortex of *Mecp2* KO mouse

The newly identified interactions between MeCP2 and multiple splicing factors prompted us to determine whether there are widespread RNA splicing changes upon loss of MeCP2. We conducted high-throughput sequencing of RNA (RNA-Seq) from the cortex of wild type and *Mecp2* knockout (KO) mice. As a measure of the quality of the RNA-Seq data, we first examined whether our data reflect transcriptional changes consistent with previous findings. We examined transcriptional changes in our RNA-Seq data by applying a negative binomial model in edgeR[34]. Recently, a meta-analysis of transcriptional changes across multiple brain regions in *Mecp2* KO or overexpression (OE) mouse identified 466 MeCP2-repressed genes based on high degree of consistency (log_2_FC > 0 in KO or log_2_FC < 0 in OE in at least 7 out of 8 datasets; FC: fold change)[9]. Of these genes, 315 genes (~68%) were also found to be up-regulated (log2FC[KO/WT] > 0) in our analysis result, suggesting significant overlap between transcriptional changes identified in our study and previous studies (S2 Table). In addition, we selected seven previously known misregulated genes in *Mecp2* KO[9, 35] as well as six novel differentially expressed gene identified by our study for further validation. qRT-PCR results show that all of them show similar changes as observed in our RNA-Seq data (Pearson’s r = 0.95) (S3 Fig). Taken together, these data indicate that our RNA-Seq data are robust in identifying transcriptional changes.

**Fig 3:**
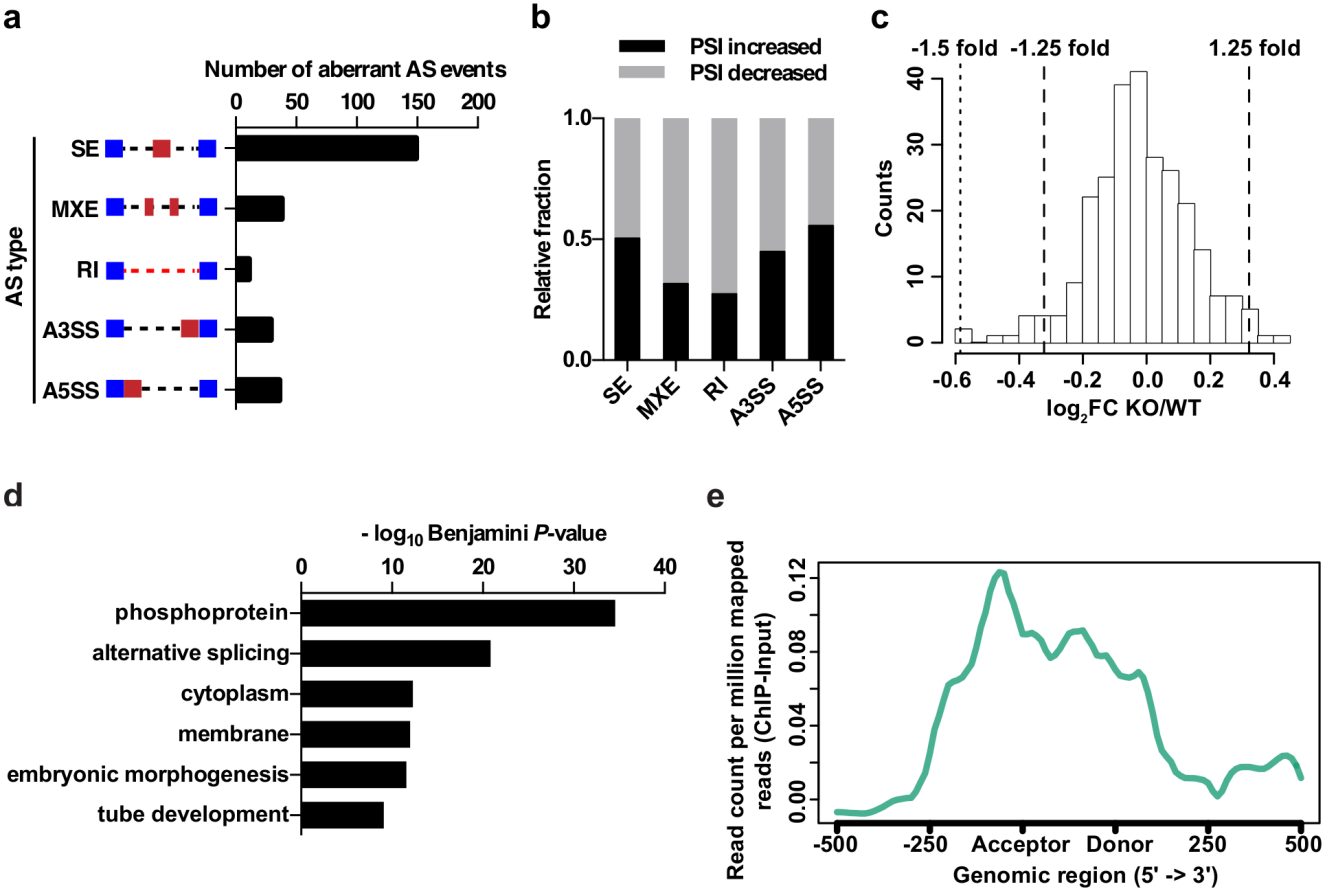
Global splicing changes in *Mecp2* KO mouse cortex and enrichment of MeCP2 around the exon/intron boundary. (a) Number of misregulated alternative splicing (AS) events by category in *Mecp2* KO cortex. (b) Relative fraction of each AS events type positively or negatively affected by loss of MeCP2. (c) Histogram of total gene expression changes in *Mecp2* KO cortex for those 232 genes associated with splicing changes in *Mecp2* KO cortex. FC: fold change. (d) Functional enrichment analysis of genes with MeCP2 peak(s) identified in ChIP-Seq data from WT mouse cortex. Top six terms are shown and –log_10_ Benjamini *P*-value is plotted for each GO term. (e) Read counts per millions of mapped reads (ChIP minus input) across regions spanning from 500bp upstream and 500bp downstream of all exons. Coverage for ChIP and input data are calculated for each biological replicate and normalized to be equal length across all the mm9 exons by sampling at equal intervals. Displayed is the average ChIP minus input profile of two biological replicates. The graph is generated using ngs.plot[41].

Next, we applied the Mixture of Isoforms (MISO)[36] algorithm to the RNA-Seq data and identified 263 alternative splicing (AS) events that were significantly changed in the cortex of *Mecp2* KO mice using a stringent filter (S3 Table; see Methods for detail). Loss of MeCP2 affects various types of AS events, including skipped exon (SE), mutually exclusive exons (MXE), retained intron (RI), alternative 5’ ss exon (A5E), and alternative 3’ ss exon (A3E) (Fig 3a). Subsequent analysis indicated that although more RI or MXE events had slightly reduced percent spliced in (PSI) value, SE, A5E and A3E events, which in total represented the majority of events, had similar number of events with increased or decreased PSI (Fig 3b). These data suggest the loss of MeCP2 affects alternative splicing in both directions, which is similar to the knockdown or overexpression of a typical splicing factor[37, 38]. Additionally, functional enrichment analysis using DAVID showed that genes with splicing changes were enriched with splice variant, alternative splicing, phosphoprotein, cell junction, compositionally biased region (Ser-rich) and plasma membrane part (S4a Fig). Interestingly, gene expression analysis on the 232 genes associated with splicing changes revealed that the majority of them have similar total expression level between WT and *Mecp2* KO (only 15 genes show larger than 1.25-fold change and only one shows larger than 1.5-fold change) (Fig 3c), suggesting that MeCP2-mediated transcriptional regulation and splicing regulation are independent of each other. To validate the splicing changes, we performed qRT-PCR with isoform specific primers to evaluate 20 SE events. We observed consistent changes in 13 genes as identified by MISO (65%), including 6 events with decreased PSI and 7 events with increased PSI in *Mecp2* KO (S5 Fig). The overall validation rate from our study of using biological replicates of tissue is comparable to the success rate using cell lines in two recent studies (74% and 71%, respectively)[39, 40], when more than 20 events were selected for validation.

To generalize our observation that loss of MeCP2 leads to global splicing changes, we analyzed RNA-Seq data generated from *Mecp2* KO hypothalamus and visual cortex in two recent studies[9, 35], respectively. Using a cutoff of |ΔPSI| ≥ 5% and Bayes factor≥1, 482 and 719 SE events were identified by MISO to be changed in *Mecp2* KO hypothalamus and visual cortex, respectively. 150 of the 482 SE events identified in the *Mecp2* KO hypothalamus and 171 of the 719 SE events identified in the *Mecp2* KO visual cortex were also found in our study (S4–S6 Tables). We focused our meta-analysis on SE events because this is the best-annotated category of alternative splicing events in the mouse genome. In summary, the large number of alternative splicing changes in independent RNA-seq data sets and the significant overlap between data sets generated from different brain regions of different lines of *Mecp2* KO mice at different ages are consistent with the notion that loss of MeCP2 results in global splicing alterations.

To further study whether MeCP2 may be directly involved in modulating splicing, we examined MeCP2 occupancy across the genome. ChIP-Seq analysis was performed using the anti-Flag antibody on chromatin prepared from the cortex of the *Mecp2-Flag* knockin mice. 20,652 high confidence MeCP2 ChIP-seq peaks were identified (S7 Table, see Methods for detailed description on ChIP-seq analysis and quality control statistics.). Based on statistical ranking and robustness of primer design, 5 of the identified peaks were selected for independent validation (highlighted in S7 Table). ChIP-qPCR on a separate cohort of *Mecp2*-Flag mice detected significant occupancy of MeCP2 at the genomic locations corresponding to these 5 peaks relative to *Gapdh* promoter (S7 Table). To gain an overall picture of MeCP2 distribution across the genome relative to genes, we examined the MeCP2 ChIP-seq signal in the 2kb region immediately upstream of all transcriptional start sites (TSS), the region from TSS to the transcription end sites (TES), and the 2kb region immediately downstream of TES across the genome. This analysis revealed that MeCP2 occupancy was depleted at promoters (~0 read count per million mapped reads [ChIP minus Input] from TSS to -2,000bp. In contrast, MeCP2 binding is enriched in the gene body (0.05–0.33 read count per million mapped reads [ChIP minus Input] from TSS to TES, S4b Fig). To assess the correlation between MeCP2 ChIP-seq signal and DNA methylation, we calculated the average percentage of mCG and mCH across all of our MeCP2 ChIP-seq peaks using previously published whole genome base-resolution methylation data in mouse cortex [42], and found that the percentage of mCG in MeCP2 ChIP-seq peaks is slightly higher than genome average (~83% vs 78%), and the percentage of mCH in MeCP2 ChIP-seq peaks is significantly higher than genome average (2.78% vs 1.30%), suggesting MeCP2 ChIP signal is correlated with mCH and mCG across the genome. These results are consistent with several previous studies that demonstrated that MeCP2 occupancy tracks DNA methylation across the genome[35, 43, 44]. Moreover, the average GC content in MeCP2 ChIP-seq peaks is ~53.8%, significantly higher the genome average of 42% [45]. The correlation between MeCP2 occupancy and GC content is consistent with findings reported in earlier this year [44]. Interestingly, gene ontology analysis found that genes with MeCP2 ChIP-seq peak(s) were enriched with GO terms of alternative splicing (Fig 3d). Indeed, alignment of MeCP2 ChIP-seq reads with the 5’ and 3’ ends of exons revealed a significant enrichment of MeCP2 ChIP-seq peaks around the exon/intron boundary and over exons (Fig 3e). Consistently, significant enrichment of hmC and mCG signals were also found at intron/exon boundary and on exons, while a modest enrichment of mCH signal was observed at the 3’ end of exons (S4c–S4e Fig). Taken together, the physical interaction between MeCP2 and splicing factors, the widespread changes in RNA splicing, and the enriched MeCP2 occupancy around exon/intron boundary are consistent with each other and strongly suggest that MeCP2 could play an important role in regulating alternative splicing.

### Altered splicing of the AMPAR genes in the cortex of *Mecp2* KO mice

**Fig 4:**
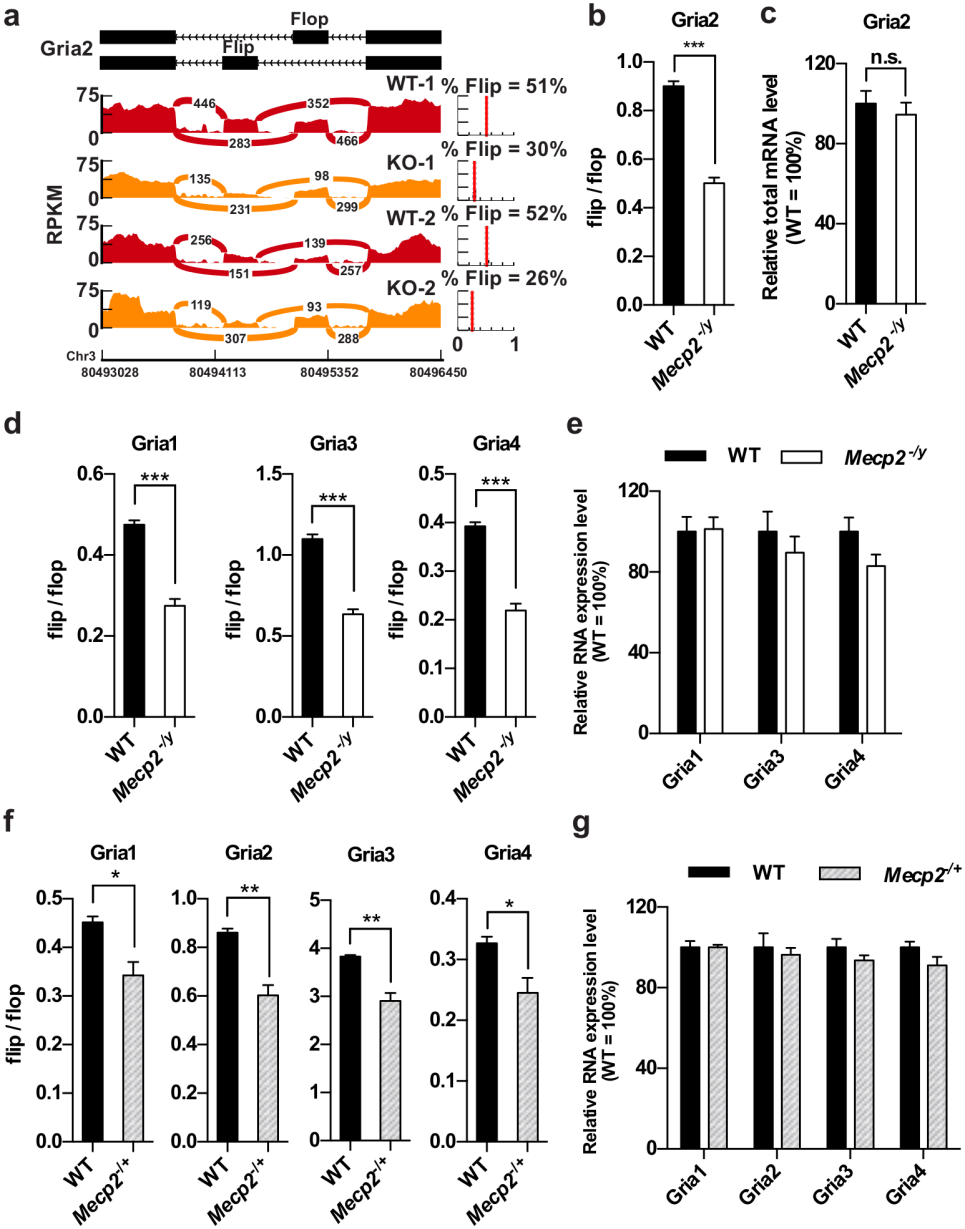
Loss of MeCP2 affects flip/flop splicing of AMPAR genes. (a) Reads distribution surrounding the flip and flop exons of *Gria2* locus from RNA-Seq data of two pairs of WT and *Mecp2* KO mouse cortex. Percentage of flip isoform (% flip) for each sample is estimated by Mixture of Isoform pipeline (MISO) and plotted on the right. (b) Quantification of flip/flop ratio of *Gria2* gene in the cortex of WT and *Mecp2* KO mice. Mean ± S.E.M is plotted. n = 8 per genotype. *** *P* < 0.001; two-tailed *t*-test. (c) Quantification of *Gria2* total mRNA level in the cortex of WT and *Mecp2* KO mice. Mean ± S.E.M is plotted. n = 8 per genotype. (d) Quantification of the flip/flop ratio of *Gria1*, *Gria3* and *Gria4* in the cortex of WT and *Mecp2* KO mice. Mean ± S.E.M is plotted. n = 8 per genotype. *** *P* < 0.001; two-tailed *t*-test. (e) Quantification of *Gria1*, *Gria3 and Gria4* total mRNA level in the cortex of WT and *Mecp2* KO mice. Mean ± S.E.M is plotted. n = 8 per genotype. (f) Quantification of the flip/flop ratio of *Gria1*, *Gria2*, *Gria3* and *Gria4* in the cortex of WT and *Mecp2*^-/+^ female mice. Mean ± S.E.M is plotted. n = 4 per genotype. * *P* < 0.05, ** *P* < 0.01; two-tailed *t*-test. (g) Quantification of *Gria1*, *Gria3 and Gria4* total mRNA level in the cortex of WT and *Mecp2*^-/+^ female mice. Mean ± S.E.M is plotted. n = 4 per genotype.

Gria2 is a major component of the AMPA receptor (AMPAR), which mediates the vast majority of fast synaptic transmission in the CNS. Two electrophysiologically distinct isoforms for Gria2 are generated by a mutually exclusive splicing event of the *Gria2* pre-mRNA. Depending of the usage of either the flip or the flop exon, *Gria2* pre-mRNA can be spliced into either the flip or the flop isoform. Our RNA-Seq data revealed that ~ 51% of all *Gria2* transcripts contained the flip exon in wild type mice. In contrast, only ~ 28% *Gria2* transcripts included the flip exon in the *Mecp2* KO mice (Fig 4a). qRT-PCR analysis in a separate cohort of animals confirmed a shift of flip/flop ratio toward a flop dominant state in *Mecp2* KO mice, while the total expression level of the *Gria2* gene remained unchanged (Fig 4b and 4c). The reduction of flip/flop ratio in *Mecp2* KO mice are not likely due to delayed development of the brain because the flip isoform is more abundant during early brain development and the flop isoform gradually increases to a comparable level of the flip isoform toward adulthood[46].

Since alternative splicing of flip/flop exons is a common feature in all AMPAR genes, we asked whether similar changes also occurred in the *Gria1*, *Gria3*, and *Gria4* genes. Quantification result showed that flip/flop ratio of *Gria1*, *Gria3*, and *Gria4* genes was significantly reduced in the cortex of *Mecp2* KO mice (Fig 4d), implicating a biased usage of flop exon in the mature transcripts of all AMPAR genes. Importantly, the total mRNA level of *Gria1* was unchanged and only subtle trend of decreasing *Gria3* and *Gria4* mRNA level was observed in *Mecp2* KO mice (Fig 4e).

Interestingly, analysis of RNA-Seq data from visual cortex and hypothalamus of *Mecp2* KO mice also showed that percentage of flip isoform is significantly decreased (S8 Fig). Note that these two studies used the Bird allele (*Mecp2*^tm1.1Bird^) and our data was generated from the Jaenisch allele (*Mecp2*^tm1.1jae^). The consistent flip/flop splicing changes across different brain regions from different knockout mouse lines suggested that reduction of flip/flop ratio is a common defect due solely to loss of MeCP2. More importantly, we also found that the percentage of flip isoform in the hypothalamus of *Mecp2* OE mice was significantly increased, which is opposite to the changes in *Mecp2* KO (S8 Fig). Together, these results strongly suggest that MeCP2 directly modulates the regulation of *Gria2* flip/flop splicing.

Finally, we tested whether splicing alteration of AMPAR genes also occurs in the cortex of heterozygous female *Mecp2*^*-/+*^ mice. Although not as drastic as that observed in *Mecp2* KO male mice, *Mecp2*^*-/+*^ mice also displayed a significant reduction of flip/flop ratio in *Gria1*, *Gria2*, *Gria3*, and *Gria4* (Fig 4f). Similar to *Mecp2* KO male mice, *Mecp2*^*-/+*^ mice had unchanged total mRNA level in all four AMPAR genes (Fig 4g). These data suggest similar change in flip/flop usage may exist in female RTT patients.

### LEDGF is involved in the regulation of Gria2 flip/flop splicing

**Fig 5:**
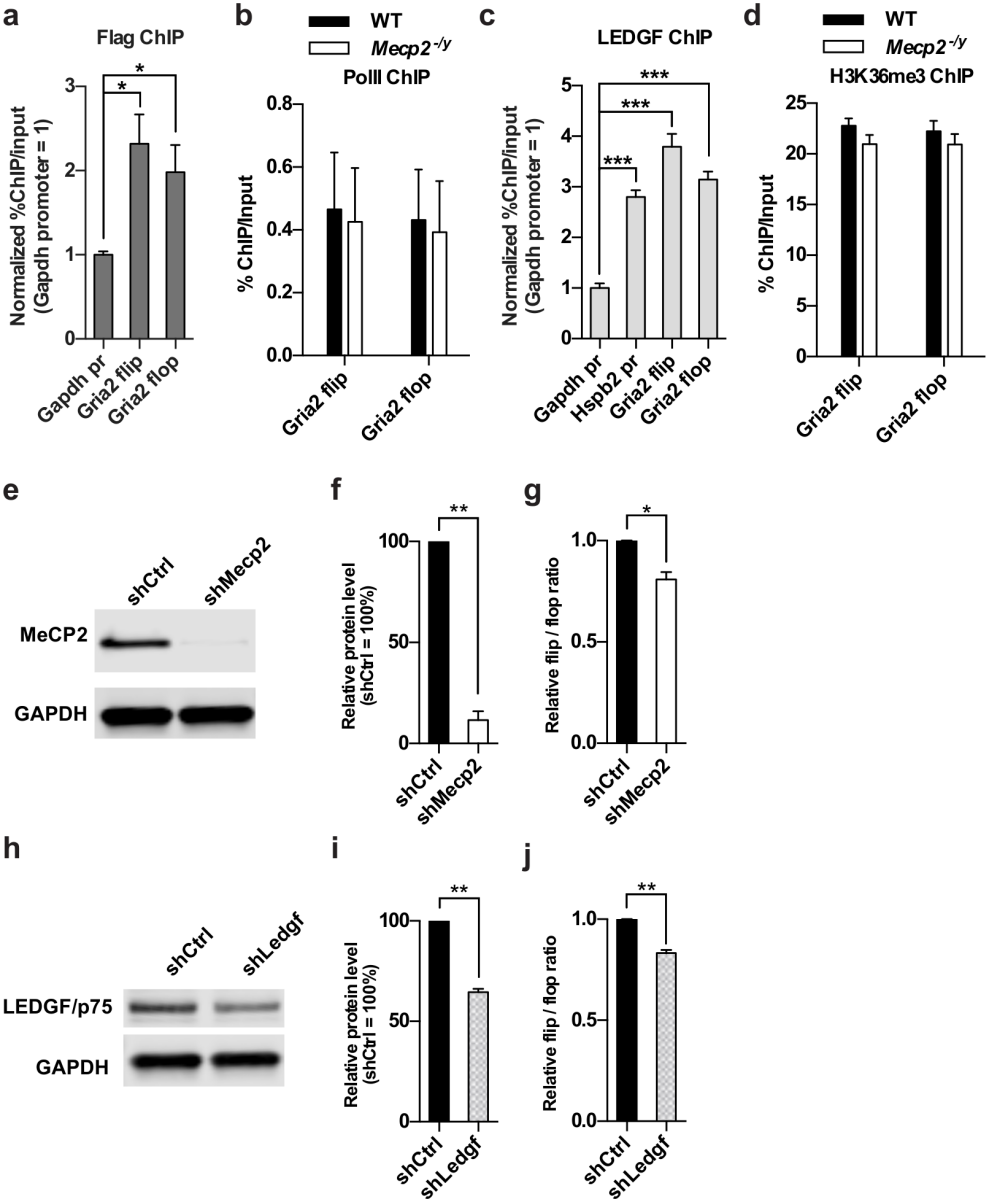
LEDGF is involved in regulation of Gria2 flip/flop splicing. (a) ChIP-qPCR analysis of MeCP2 occupancy on *Gapdh* promoter, *Gria2* flip and flop exon in *Mecp2-Flag* mouse cortex. pr: promoter. Mean ± S.E.M is plotted. n = 4 per genotype. * *P* < 0.05; one-way ANOVA followed with Holm-Sidak’s multiple comparisons test. (b) ChIP-qPCR analysis of PolII occupancy on *Gria2* flip and flop exon in WT and *Mecp2* KO cortex. Mean ± S.E.M is plotted. n = 4 per genotype. (c) ChIP-qPCR analysis of LEDGF occupancy on *Gria2* flip and flop exon in WT mouse cortex. Mean ± S.E.M; n = 5; *** *P* < 0.001; one-way ANOVA followed with Holm-Sidak's multiple comparisons test. (d) ChIP-qPCR analysis of H3K36me3 occupancy on *Gria2* flip and flop exons in WT and *Mecp2* KO cortex. n = 4 per genotype. (e-g) Representative Western blot analysis of MeCP2 (e), quantification of MeCP2 protein level (f), and flip/flop ratio (g) in Neuro-2A cells co-transfected with *Mecp2* shRNA plasmid, MeCP2 expression plasmid and Gria2 minigene plasmid. Mean ± S.E.M of three independent experiments, * *P* < 0.05, *** *P* < 0.001; two-tailed *t*-test. (h-j) Representative Western blot analysis of LEDGF (h), quantification of LEDGF protein level (i), and flip/flop ratio (j) in Neuro-2A cells co-transfected with *Ledgf* shRNA plasmid and Gria2 minigene plasmid. Mean ± S.E.M of three (i) or four (j) independent experiments, ** *P* < 0.01; two-tailed *t*-test.

To determine how loss of MeCP2 affects the splicing of flip/flop exon, we focused on the *Gria2* gene to explore the potential involvement of several recent models of splicing regulation. Modulation of PolII elongation rate has been proposed as one model of how epigenetic mechanisms influence splicing. Slow PolII elongation rate allows longer time for spliceosome to assemble and hence increase the chance of the alternative exon being included in the mature transcript[47]. A recent study suggested that MeCP2 is enriched in particular alternative exons and facilitates exon inclusion by pausing PolII in cultured cells[48]. We set out to test whether MeCP2 regulates *Gria2* flip/flop splicing through similar mechanism in the brain. Chromatin immunoprecipitation (ChIP) followed by qRT-PCR showed a significant enrichment of MeCP2 on the flip and flop exons of *Gria2* gene (Fig 5a). However, no significant difference in PolII occupancy on the flip and flop exons between the wild type and *Mecp2* KO mice was found by PolII ChIP (Fig 5b), suggesting the involvement of a PolII-independent mechanism underlying the flip/flop splicing change in *Mecp2* KO brain. Another interesting epigenetic model for alternative splicing regulation is that histone modification can be bound by adaptor proteins which in turn recruit specific splicing factor to alternative exons[47]. It has been previously shown that trimethylated histone H3 lysine 36 (H3K36me3) is enriched on exons and can be bound by LEDGF, which recruits splicing factors such as SRSF1 to regulate splicing[49]. Although significant LEDGF occupancy was detected on the flip and flop exons (Fig 5c), no significant difference in the occupancy of H3K36me3 on the flop and flip exons was detected between the wild type and *Mecp2* KO brain (Fig 5d).

To determine whether LEDGF is functionally involved in the regulation of Gria2 flip/flop splicing, we tested the effect of knockdowning LEDGF on flip/flop ratio in a neuroblastoma cell line, Neuro-2A, using a *Gria2* minigene. The *Gria2* minigene spans the genomic region from exon 13 to exon 15 of *Gria2* (S9a Fig, either the flip or flop exon can be included as exon 14). As a control, we co-transfected *Mecp2* shRNA, Gria2 minigene along with a MeCP2 overexpression plasmid into Neuro-2a cells and found that flip/flop ratio is significantly reduced upon *Mecp2* knockdown (Fig 5e and 5f), indicating that this artificial assay is capable of discovering factors that potentially affect flip/flop splicing. Next, we transfected a *Ledgf* shRNA in the cells and tested its effect on flip/flop splicing. Similar to *Mecp2* knockdown, *Ledgf* knockdown also leads to a reduction of flip/flop ratio (Fig 5g and 5h), suggesting that LEDGF is functionally involved in the regulation of Gria2 flip/flop splicing.

### Functional link between altered flip/flop splicing and synaptic phenotypes in the *Mecp2* KO mice

**Fig 6:**
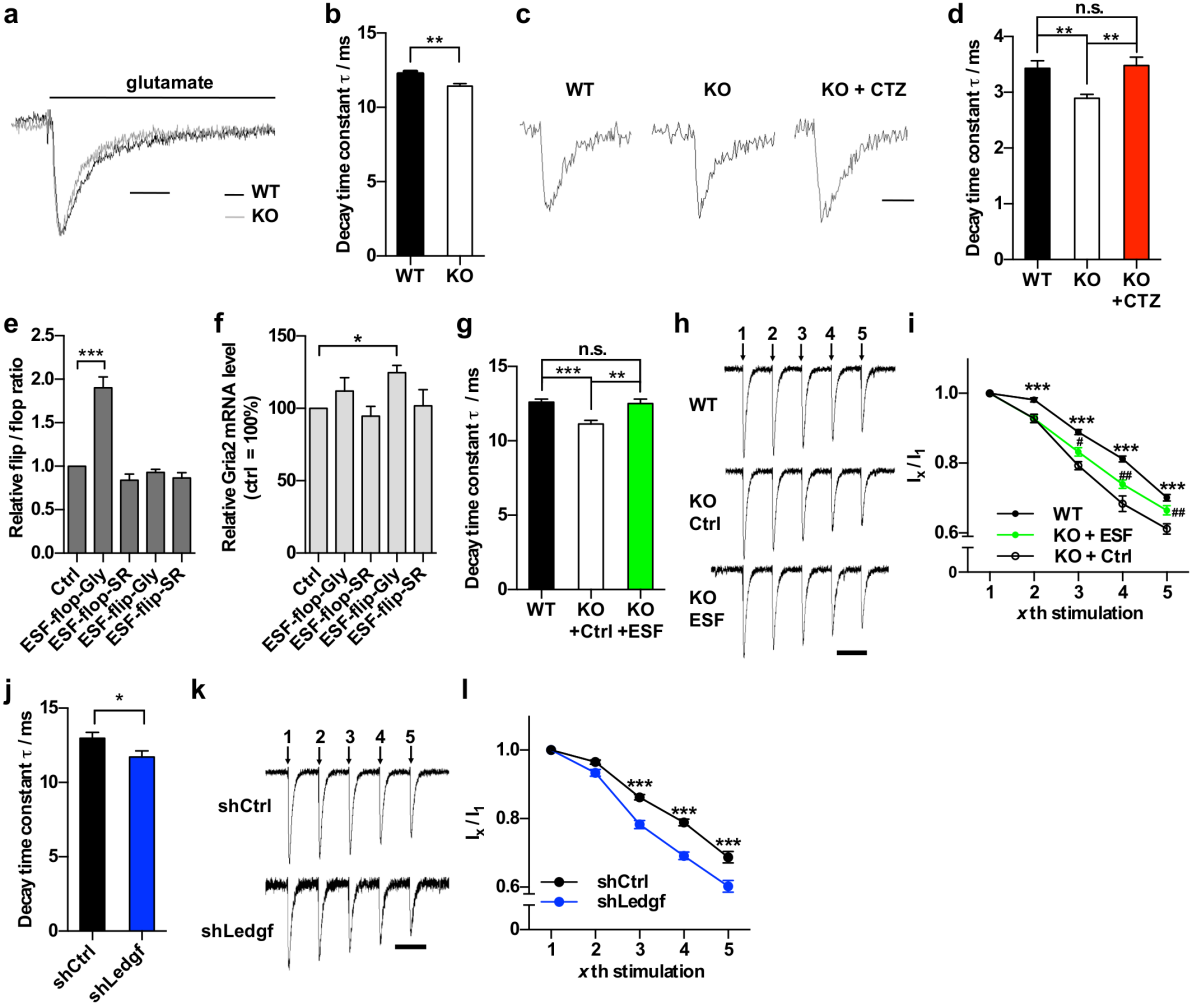
Altered AMPA receptor decay kinetics and synaptic transmission in *Mecp2* KO cortical neurons can be reversed by ESF. (a) Representative sample trace of glutamate-evoked current in WT and *Mecp2* KO cortical neurons. Scale bar = 15ms. (b) Quantification of decay time constant τ of glutamate-evoked current in WT and *Mecp2* KO cortical neurons. Mean ± S.E.M is plotted. n = 37 for WT, n = 33 for KO. ** *P* < 0.01; two tailed *t*-test. (c) Representative mEPSC sample trace in WT and *Mecp2* KO cortical neurons with or without CTZ treatment. Scale bar = 10ms. (d) Quantification of decay time constant τ of mEPSC. Mean ± S.E.M is plotted. n = 42 for WT, n = 46 for KO, n = 38 for KO + CTZ. ** *P* < 0.01; one-way ANOVA with Tukey’s multiple comparisons test. (e-f) qRT-PCR analysis the flip/flop ratio (e) and total *Gria2* minigene (f) expression in HEK 293 cells co-transfected with indicated ESF and the *Gria2* minigene. Mean ± S.E.M is plotted. n = 4 for each group. * *P* < 0.05, *** *P* < 0.001; two tailed *t*-test. (g) Quantification of decay time constant τ of glutamate-evoked current in WT, *Mecp2* KO (KO+Ctrl) and *Mecp2* KO cortical neurons infected with ESF-flop-Gly lentivirus (KO+ESF). Recording was done on acute slice from mouse infected with lentivirus two weeks after stereotaxic injection. Mean ± S.E.M is plotted. n = 50 for WT, n = 26 for KO+Ctrl, n = 27 for KO+ESF. ** *P* < 0.01, *** *P* < 0.001; one-way ANOVA with Tukey’s multiple comparisons test. (h) Representative sample trace of AMPAR-mediated current in response to repetitive stimulations in WT and *Mecp2* KO neurons infected with control or ESF-flop-Gly lentivirus (KO+Ctrl or KO+ESF). Scale bar, 100ms. (i) Relative current amplitude of the xth stimulation to first stimulation in WT and *Mecp2* KO neurons infected with either control or ESF-flop-Gly lentivirus (KO+Ctrl or KO+ESF). Mean ± S.E.M is plotted. n = 36 for WT, n = 18 for KO+Ctrl, n = 18 for KO+ESF. Asterisk denotes *P*-value for comparison between WT and KO+Ctrl, *** *P* < 0.001. Pound sign denotes *P*-value for comparison between KO+ESF and KO+Ctrl, # *P* < 0.05, ## *P* < 0.01; repeated measures two-way ANOVA with Tukey’s multiple comparisons test. (j) Quantification of decay time constant τ of glutamate-evoked current in neurons infected with shCtrl or shLedgf lentivirus. Mean ± S.E.M is plotted. n = 13 for shCtrl, n = 12 for shLedgf, * *P* < 0.05; two-tailed *t*-test. (k) Representative sample trace of AMPAR-mediated current in response to repetitive stimulations in neurons infected with shCtrl or shLedgf lentivirus. Scale bar, 100ms. (l) Relative current amplitude of xth stimulation to first stimulation in neurons infected with shCtrl or shLedgf. Mean ± S.E.M is plotted. n = 12 for each group, *** *P* < 0.001; repeated measures two-way ANOVA with Tukey's multiple comparisons test.

Flip/flop exon encodes a 38 amino acids sequence in the ligand binding domain of AMPARs that controls desensitization rate. Compared to flip-containing receptors, flop-containing receptors desensitize with faster kinetics[50, 51]. To determine the functional consequence of altered flip/flop splicing in the cortex of *Mecp2* KO mice, we performed outside-out patch clamp recording of glutamate-evoked current on layer 2/3 pyramidal neurons in acute brain slices. We found that the decay time constant τ was significantly reduced in *Mecp2* KO mice (Fig 6a and 6b). In addition to evoked response, regular whole cell patch clamp recording of spontaneous synaptic events also detected a faster decay in miniature excitatory postsynaptic current (mEPSC) in layer 2/3 pyramidal neurons from the *Mecp2* KO mice. Finally, bath application of cyclothiazide (CTZ), a positive allosteric modulator of AMPARs that inhibits desensitization of AMPARs[52], slowed down the decay kinetics in *Mecp2* KO slice to a comparable level of wild type cells (Fig 6c and 6d). Together, these results uncover a previously unappreciated defect of faster desensitization kinetics of AMPAR-gated current in the *Mecp2* KO mice, which correlates with altered flip/flop splicing and can be modulated by pharmacological reagents.

To causally link the change in flip/flop splicing and the altered AMPAR desensitization kinetics, we used engineered splicing factors (ESF)[53, 54] to specifically manipulate flip/flop splicing in the brain of *Mecp2* KO mice. ESF is composed of a sequence-specific RNA-binding domain derived from human Pumilio1 (PUF domain) and a functional domain that suppresses (Gly domain) or enhances (SR domain) inclusion of a specific exon. We evaluated the effect of four ESFs (ESF-flop-Gly [flop suppressor], ESF-flop-SR [flop enhancer], ESF-flip-Gly [flip suppressor] and ESF-flip-SR [flip enhancer]) on flip/flop splicing using a *Gria2* minigene (S9a Fig). We found that ESF-flop-Gly significantly increased the flip/flop ratio (Fig 6e), an effect opposite to the change we observed in the cortex of *Mecp2* KO mice. Moreover, ESF-flop-Gly didn’t change the level of total *Gria2* minigene (Fig 6f).

To further test the effect of ESF-flop-Gly on flip/flop splicing of the endogenous *Gria2* transcript in neurons, we infected primary cortical neurons with adeno-associated virus (AAV) encoding either mCherry alone or ESF-flop-Gly and mCherry. As expected, AAV-ESF-flop-Gly-mCherry significantly altered the flip/flop splicing balance to favor the use of the flip exon (S9b and S9c Fig), suggesting that ESF-flop-Gly could be used *in vivo* to reverse the flip/flop splicing defect in *Mecp2* KO mice. To that end, we injected lentivirus expressing ESF-flop-Gly into the cortex of *Mecp2* KO mice, and measured the decay time constant τ of glutamate-evoked AMPAR-gated current in the outside-out patch clamp mode in acute brain slices 2 weeks post injection. Compared to neurons infected with control virus (KO+Ctrl), ESF-flop-Gly expressing neurons (KO+ESF) had a significant larger decay time constant τ, which was indistinguishable from that of WT cells (Fig 6g). These results strongly suggest that altered flip/flop splicing is required for a specific synaptic phenotype in the *Mecp2* KO mice.

To further examine the effect of altered flip/flop splicing on synaptic transmission, we applied repetitive stimulation on the neurons in an interval of 100 ms and recorded the AMPAR-gated current. Upon repetitive stimulation, a fraction of AMPA receptors desensitizes and the short interval between stimulation does not allow full recovery. As a result, fewer AMPA receptors can respond to the subsequent stimulation and therefore current diminished. Comparing to WT neurons, KO neurons displayed even more drastic decrease in current amplitude over the course of five stimulations (Fig 6h and 6i). This difference could be partially due to a higher percentage of flop isoform that are more easily desensitized in the KO neurons. Consistent with this hypothesis, overexpressing ESF-flop-Gly in KO neurons partially rescued this phenotype (Fig 6h and 6i). These data suggest the altered flip/flop splicing ratio has important impact on synaptic transmission in the *Mecp2* KO cortex, which can be reversed by ESF designed to specifically target flip/flop exons.

To determine the functional outcome of *Ledgf* knockdown-induced change in *Gria2* flip/flop splicing, we injected lentivirus encoding shLedgf and control shRNA into the cortex of wild type mice. We found that *Ledgf* knockdown resulted in a significantly reduced decay time constant τ of glutamate-evoked AMPAR-gated current (Fig 6j), an effect similar to that caused by the loss of MeCP2 (Fig 6a and 6b). In addition, *Ledgf* knockdown led to significantly weaker response upon repetitive stimulations (Fig 6k and 6l), another phenotype caused by the loss of MeCP2 (Fig 6h and 6i). These data correlate well with the *Gria2* minigene assay in Fig 5 and further support that both LEDGF and MeCP2 are required for the normal splicing of *Gria2* flip/flop exons.

## Discussion

MeCP2 has been previously implicated in regulating alternative splicing of RNA in two studies. In 2005, Young et al reported RNA-dependent interaction between MeCP2 and YB1 in a neuroblastoma cell line forced to overexpress MeCP2 and some changes in alternative splicing in the *Mecp2*^*308/y*^ brain[23]. In 2013, Maunakea et al reported intragenic DNA methylation-dependent MeCP2 binding to alternatively spliced exons in cancer cell lines[48]. Our work substantially extends these previous studies in several ways, and to our knowledge, this is the first report of the functional consequence for MeCP2-mediated splicing.

First, the physical interaction between MeCP2 and its interacting partners identified in our study are independent of any nucleic acid, suggesting that MeCP2 does not need to bind to RNA in order to regulate splicing. Additionally, these physical interactions have more physiological relevance, because they were identified in the mouse brain where MeCP2 is expressed from its endogenous locus. Furthermore, we identified multiple splicing factors as novel MeCP2-interacting partners in the brain. Since these factors are not part of the core splicing machinery but rather affect splicing as accessory splicing factors[55], the biochemical mechanism underlying their involvement in splicing regulation is not well known. Their interaction with MeCP2, a known chromatin protein, provides novel clues for studying how these factors regulate splicing. In addition, because we used a different RTT mouse model (*Mecp2* KO mice in our study vs. *Mecp2*^*308/y*^ mice in Young et al[23]) and a more sensitive method to profile alternative splicing (RNA-seq vs. microarray), the altered splicing events identified in our study were different from those previously identified by Young et al[23]. Nonetheless, combining results from three independent unbiased approaches (Co-IP mass spectrometry, RNA-seq and ChIP-seq), our study provides strong evidences for a significant involvement of MeCP2 in regulating RNA splicing.

Second, we discovered significant MeCP2 occupancy around exon/intron boundary and exons in the mouse brain, and characterized gene exon specific interaction between MeCP2 and two splicing regulators, providing a potential mechanism for MeCP2-dependent splicing regulation. Recent evidence suggests intragenic DNA methylation recruits MeCP2 and regulates pre-mRNA splicing through altering DNA polymerase II elongation rate[48]. However, our data suggests that it is not responsible for the altered flip/flop splicing in the cortex of *Mecp2* KO mice. Instead, our results suggest a new model that co-occupancy of MeCP2 and LEDGF on the chromatin is required for the normal flip/flop splicing in the *Gria2* gene.

Finally, and most importantly, we established a functional link between specific splicing changes caused by the loss of MeCP2 function to synaptic changes in RTT mice. The fact that a ESF specifically rescues the flip/flop splicing defect can reverse the corresponding synaptic changes in RTT brain strongly suggest that the specific change in synaptic property (AMPAR kinetics) is caused by altered flip/flop splicing. Given the central role of AMPARs in synaptic transmission, it is likely the altered AMPAR kinetics will lead to altered synaptic functions other than the repetitive stimulation paradigm employed in our study. Future study is needed to mechanistically link the altered AMPAR kinetics with specific neuronal defects in RTT symptoms, and to evaluate the effect of reversing flip/flop splicing on RTT disease progression. In addition to the flip/flop choice in AMPARs, alterative splicing of several other genes (e.g. *Nrxn1*, *Dscam*, *lin7a*) that play important roles in synaptic functions were changed in the RTT mouse cortex, indicating that additional synaptic changes may be caused by splicing deficits. Thus, altered RNA splicing appears to be a novel molecular mechanism underlying synaptic dysfunction in RTT.

Splicing misregulation has been increasingly recognized as a significant contributor to a number of neurological diseases, such as SMA[56], FTDP-17[27], ALS[57] and myotonic dystrophy[58]. The mechanistic study of how the genes mutated in neurological diseases can directly affect alternative splicing, as well as the functional consequences of splicing alteration in such diseases, will have important implications in human health. Our study adds to the growing list of studies on the novel links between specific events of altered splicing and neurological diseases.

## Materials and Methods

### Ethics statement

All animal procedures were performed according to protocols approved by the Institutional Animal Care and Use Committee at the University of Wisconsin-Madison. All mice in this study were euthanized by CO2 asphyxiation, according to the guidelines of the RARC at the University of Wisconsin-Madison and the recommendations of the Panel on Euthanasia of the American Veterinary Association.

### Animals

The *Mecp2*-Flag mice have a Flag sequence inserted intermediately before the stop codon of the *Mecp2* locus[34]. The *Mecp2* KO mice used in this study are the Jaenisch strain (*Mecp2*^tm1.1jae^)[59]. Mice were housed in a facility with 12-hr light/12-hr dark cycle.

### Plasmids

pRK5-HA-MeCP2-WT, Δ1–77, Δ1–162, Δ78–162 and Δ381–492 were a gift from Dr. Zilong Qiu[25]. DNA encoding Δ163–380, MeCP2^R168X^, MeCP2^R255X^, MeCP2^R270X^ and MeCP2^R290X^ were PCR amplified and inserted into pRK5-HA by replacing the sequence between SalI site and NotI site of pRK5-HA-MeCP2 using Gibson cloning (NEB). To construct Myc-tagged protein expression plasmid, cDNA of LEDGF/p52, LEDGF/p75, and DHX9 were amplified from a mouse cortex cDNA library using a Myc sequence-containing primer and inserted into pRK5 backbone. LEDGF is also known as Psip1. Engineered splicing factor (ESF) were designed to target the Flip (GCCAAGGA) and the Flop (GCAGCGGG) exons[38]. Gria2 minigene was constructed by amplifying *Gria2* Exon13 to Exon15 from mouse genomic DNA and inserted into pEGFP-C1 backbone. The *Ledgf* shRNA (shLedgf) target sequence (5’-GCA GCT ACT GAA GTC AAG ATT C-3’) was adapted from a previous study[60] and cloned into pLL3.7 backbone. The *Mecp2* shRNA (shMecp2) construct was used in our previous study[61]. A scrambled sequence (5’-GGA ATC TCA TTC GAT GCA TAC-3’) was used as negative control (shCtrl).

### Co-immunoprecipitation and mass spectrometry

Nuclei were extracted from the whole brain of WT and *Mecp2-Flag* mice as previously described[62]. Purified nuclei were resuspended in lysis buffer containing 20mM Tris, 150mM NaCl, 1.5mM MgCl_2_, 1mM EDTA, 10% Glycerol, 0.2% NP-40 and 1X proteinase inhibitors cocktail (Roche) and sonicated using a Misonix 3000. After centrifuging at 20,000g for 20min at 4°C, supernatant was incubated with 50ul of Anti-Flag M2 Magnetic Beads (Sigma) overnight at 4°C. In the following day, beads were washed with lysis buffer for 6 times. Bound protein was eluted by competition with 100 mg/ml of Flag Peptide (Sigma F3290). Eluted proteins from 5 IPs per genotype were pooled together and precipitated by adding 8 volume of pre-chilled acetone. Pellet was resuspended in 100mM Ammonium Bicarbonate solution. After DTT and IOAA treatment, protein was digested into peptides using Trypsin Gold (Promega) and Proteinase Max (Promega) overnight at 37°C. Peptides were separated by a nano HPLC and analyzed by a Thermo LTQ mass spectrometer. MS/MS spectra data was analyzed using Bioworks software (Thermo). Only proteins identified in Flag IP eluate from *Mecp2-Flag* mice but not WT mice were considered to be potential MeCP2-interacting proteins.

### Co-immunoprecipitation and western blot

Co-IP was performed as described above except using Dynabeads (Life Technologies). For co-IP with Benzonase treatment, lysate was treated with 250 Unit of Benzonase per mouse brain for 1hr at 4°C before incubating with beads. Proteins were eluted by adding 1X LDS sample buffer (Life Technologies) and heated at 95°C for 10min.

Proteins were resolved in a 10% SDS-PAGE gel and transferred into a nitrocellulose membrane. Membrane was blocked with 5% non-fat milk in PBS for 1 hour followed by incubating with primary antibody overnight at 4°C. Membrane was washed 3 times with PBST and incubated with DyLight Fluor Secondary Antibodies (Pierce) for one hour at room temperature. Membrane was imaged on a LI-COR Odyssey Imager. Western blot quantification was done using ImageJ. Primary antibodies used in this study were: anti-DHX9 (Abcam ab26271, 1:2000), anti-FLAG (Sigma M2, 1:1000), anti-FUS (Bethyl A300-293A, 1:10000), anti-HA (Covance MMS-101P, 1:5000), anti-hnRNP F+H (Abcam ab10689, 1:3000), anti-LEDGF (Bethyl A300-847A, 1:1500), anti-MeCP2 (Abcam ab50005, 1:2000), anti-Myc (Cell signaling 71D10, 1:1000), and anti-TDP-43 (ProteinTech 10782-2-AP, 1:1000).

### Transfection and co-immunoprecipitation

HA-MeCP2 construct was co-transfected with Myc-LEDGF or Myc-DHX9 (1:1 ratio) into HEK293 cells using GenJet transfection reagent (Signagen). 24 hours after transfection, cells were washed with PBS twice and directly lysed with Pierce IP Lysis Buffer (Thermo Scientific) for 10min on ice. Lysate was centrifuged at 16,000g for 10min at 4°C and pellet was discarded. Six hours before lysate preparation, 30ul Dynabeads protein G was incubated with 3ug of anti-HA (Covance) or anti-Myc (Millipore) at 4°C to form the antibody-proteinG-bead complex. After washing off excess antibody, beads were incubated with lysate overnight at 4°C. Beads were washed with lysis buffer 6 times and then eluted by adding 1X LDS sample buffer (Life Technologies) and heated at 95°C for 10min.

### RNA-Seq analysis

Total RNA was extracted from cortices of 6-weeks-old WT and *Mecp2* KO mice using Qiagen RNeasy Mini Plus kit. Genomic DNA was removed by a gDNA Eliminator column. 150ng total RNA was used to prepare sequencing library according to manufacturer’s instructions (Nugen Encore Complete). Each Library was subject to one lane of 100bp single end sequencing using Illumina Hi-Seq 2000. Reads were mapped to the mouse genome (mm9) using Tophat (2.0.8). Reads count for each gene was calculated using htseq-count function in the HTSeq package. Differential gene expression analysis was done using edgeR in R. Splicing analysis was performed using the Mixture of Isoforms pipeline (MISO 0.4.7). Considering the high similarity of the two replicates for each genotype (Correlation = 0.98 for each), reads from two replicates were combined for each genotype and processed with MISO. A stringent filter (total reads for the event ≥ 1000, reads supporting inclusion or exclusion isoform ≥ 50, total reads supporting inclusion and exclusion isoform ≥ 100, |ΔPSI| ≥ 0.20 and Bayes-factor ≥ 20) was used to generate a list of differential splicing events. Read density plot was generated using sashimi plot built in MISO.

RNA-Seq data from Chen et al[35] and Gabel et al[9] were processed as above for splicing analysis. A less stringent filter (total reads for the event ≥ 20, |ΔPSI| ≥ 0.05 and Bayes-factor ≥ 1) was applied to allow for generating more events for further overlap analysis.

### Gene Ontology analysis

Gene Ontology (GO) analysis was done using DAVID[63]. Briefly, official gene symbols were submitted to DAVID. We used our own RNA-seq data and applied a cutoff of RPKM ≥ 0.5 to generate a list of genes expressed in the mouse cortex (13846 genes). This set of genes expressed in the mouse cortex was used as background for all GO analysis in this manuscript. Terms with Benjamini adjusted *P*-value < = 0.05 was considered as significant.

### RNA extraction and qRT-PCR

Total RNA was extracted from cortices of 6-8-week-old wild type (WT) and *Mecp2* KO male mice or 15-18-month-old WT and *Mecp2* KO female mice using Qiagen RNeasy Mini Plus kit with on-column DNase treatment. RNA extraction from HEK293 or N2A cells was performed using TRIzol (Life Technology). RNA was reverse transcribed into cDNA using qScript cDNA SuperMix (Quanta Biosciences). qPCR was performed on an ABI Step-One plus machine using SYBR Green qPCR Master Mix (Biotool). *Gapdh* was used as endogenous control and 2^−ΔCt^ method was used to calculate fold change. See S8 Table for primer sequence.

### Chromatin immunoprecipitation

Chromatin immunoprecipitation (ChIP) was performed as previously reported[29]. Briefly, cortex tissue was dissected from 6-8-week-old mice, minced and crosslinked in 1% formaldehyde (wt/vol) and sonicated using a Misonix 3000. Antibody was first bound to Dynabeads and then incubated with sheared chromatin overnight at 4°C. After 4 washes with RIPA buffer and 1 wash with TE buffer, bound chromatin was eluted and reverse crosslinked at 65°C overnight. Eluted DNA was treated with RNase A (Thermo Scientific) and proteinase K (Promega), purified by phenol-chloroform extraction and dissolved in water. Antibodies used were: anti-Flag (Sigma M2), anti-LEDGF (Bethyl A300-847A), anti-H3K36me3 (Abcam ab9050), and anti-PolII (Abcam ab5408). Primer sequence for ChIP-qPCR is provided in S8 Table.

### ChIP-seq data analysis

ChIP-Seq data were generated from two biological replicates (referred to as WT1 and WT2). Raw data was aligned to the mouse genome version mm9 with Bowtie (0.12.7). After excluding non-mapping reads, we had 72, 221, 924 reads for WT1 ChIP and 31, 333, 769 for its input and 84, 871, 157 reads for WT2 ChIP and 22, 412, 408 for its input. We firstly evaluated the quality of these data with respect to ENCODE’s ChIP-seq quality control metrics[64]. The Normalized Strand Cross Correlation (NSC) for WT1 ChIP and WT2 ChIP is 1.3 and 1.4, respectively. Another quality control measure is PCR Bottleneck Coefficient (PBC), which gives an estimate of the complexity of the ChIP-seq library[65]. PBC<0.5 indicates PCR bottlenecks are present in sequenced libraries. The PBC ranged within [0.63 0.83] across WT1 ChIP sample and [0.85, 0.94] for the WT1 input sample. Similarly, the PBC ranged within [0.63 0.83] across WT2 ChIP sample and was 0.93 for the WT2 input sample. These numbers suggest our libraries were of good quality.

We carried out peak calling using MOSAiCS package in R[66] using default parameters except for fdrRelaxed = 0.1 for WT1 and WT2 and fdrRelaxed = 0.2 for pooled replicates. Bin and fragment sizes were set to 200 bps for all the runs. We followed a conservative strategy and obtained peaks for individual replicates at false discovery rate of 0.1 and for pooled sample run at 0.2. Then, we identified the peaks in the intersection of the three peak lists and filtered them with mosaics parameters: logMinP > = -log_10_(0.05) & peakSize > = 150 & aveLog2Ratio > = log_2_(1.5). This resulted in a total of 20, 652 peaks with median size of 1731 bps. We performed location analysis using mm9 Refseq genes and the nomenclature in Blahnik et al[67].

### Analysis of the level of CG methylation, CHG methylation, and CHH methylation in MeCP2 ChIP-Seq peaks

The previously published independent datasets were used[42]. DNA methylation data in the frontal cortex of adult mouse (10-wk-old) were downloaded under accession number GSM 1173784. Each context of the cytosine methylation and the two following bases from the same strand was considered independently: CG, CHG or CHH (where H = A, C or T). To determine the frequency of each context, the frequency of the cytosine methylation of each context in MeCP2 ChIP-seq peaks was estimated as the average of ratio /(*x*) = n_m_(*x*)/n_tot_(*x*), where *n*_m_(*x*) is the number of reads supporting a methylated cytosine at position *x* and *n*_tot_(*x*) is the total number of reads at that position.

### Gria2 minigene splicing assay

ESF construct was co-transfected into 293 cells with *Gria2* minigene (8:2 ratio) using GenJet transfection reagent (Signagen). To test the effect of *Mecp2* knockdown on *Gria2* splicing, shMecp2, *Mecp2* overexpression construct and Gria2 minigene (4.5:4.5:1 ratio) was co-transfected into N2A cells with using GenJet. To test the effect of *Ledgf* knockdown on *Gria2* splicing, shLedgf construct and *Gria2* minigene (9:1 ratio) was co-transfected into N2A cells using GenJet. Cells were lysed in TRIzol 48 hours after transfection for qRT-PCR analysis.

### Electrophysiology

Male mice at 4–6 weeks postnatal were used. Coronal brain slices (400 μm) were prepared in ice-cold modified artificial cerebrospinal fluid (aCSF) (in mM: 124 NaCl, 2.5 KCl, 1 CaCl_2_, 2 MgSO_4_, 1.25 NaH_2_PO_4_, 26 NaHCO_3_, and 15 glucose) bubbled with 95%O_2_/5%CO_2_. Then the slices were incubated in normal aCSF (in mM: 124 NaCl, 2.5 KCl, 2.5 CaCl_2_, 1.2 MgSO_4_, 1.25 NaH_2_PO_4_, 25 NaHCO_3_, and 15 glucose) at room temperature for at least 1 hour and then transferred to a submerged recording chamber perfused with 95%O_2_/5%CO_2_ saturated aCSF for electrophysiological recordings. Whole-cell recording of mEPSCs and outside-out patch recording of glutamate-evoked currents was performed from the Layer 2/3 pyramidal neurons at room temperature. TTX (1 μM), D-APV (20 μM), bicuculline (50 μM) were added into the perfused aCSF to block voltage gated Na^+^ channels, NMDA receptors and GABA receptors respectively. The patch pipette (3–4 MΩ) solution contained (in mM): 140 Cs-Gluconate, 7.5 CsCl, 10 HEPES, 0.5 EGTA-Cs, 4 Mg-ATP, and 0.3 Li-GTP, pH 7.4. Raw data were amplified with a Multiclamp 700B amplifier and acquired with pClamp10.2 software (Molecular Devices). Neuronal currents were recorded under voltage clamp at the holding potential of -70 mV. An ALA fast perfusion system was used to perform application of glutamate (10 mM). In some experiments, CTZ (50 μM) was added. The detection of mEPSCs and exponential fitting were performed using Clampfit 10.2. The decay of glutamate evoked currents was fitted with double-exponential functions, and the fast- and slow- time constant were obtained. Signals were filtered at 2 Hz and sampled at 10 kHz by Digidata 1440A (Molecular Devices). mEPSCs were analyzed using the Template Search tool of the Clampfit10.2. To create the template, several well-shaped mEPSCs traces were picked and averaged to the template window. The mEPSCs events were accepted manually. Amplitude and the weighted time constant of decay phase of both mEPSCs and glutamate evoked currents were acquired.

To investigate whether enhanced depression of AMPAR responses to burst-type stimulations is expressed at synapses, we recorded excitatory postsynaptic potentials evoked through a bipolar stimulating electrode (FHC Inc.) placed in the white matter (eEPSCs, five pulses at 10 Hz). AMPAR-mediated eEPSCs were recorded in the presence of D-APV (20 μM) and bicuculline (50 μM) at a holding potential of -70 mV. The data was analyzed with Clampfit 10.

### Lentivirus preparation and stereotaxic injection

Lentivirus preparation was performed as described[68] except that we use minimum amount of media (leftover in the tubes) to resuspend the virus. Stereotaxic injection was done as previously described[61].

### AAV infection

Custom AAV encoding ESF was generated by Vigene. DIV7 primary cortical neurons were infected with AAV at a MOI of 10^5^. AAV was removed 48 hours after infection and cells were collected 7 days after infection for qRT-PCR analysis.

### Statistics

No statistical procedure was used to predetermine sample size. Student’s *t*-test was used to compare means between two groups. Multiple *t*-test comparisons were corrected using Benjamini-Hochberg procedure. One-way ANOVA followed by Tukey’s multiple comparison tests was used to test difference in experiments with multiple groups. Two-way ANOVA with repeated measure followed by Bonferroni's multiple comparisons test was used for analysis in the repetitive stimulation experiments. Statistical calculation was performed using Microsoft Excel and Graphpad Prism.

## Supporting Information

S1 Fig Interaction between MeCP2 and splicing factors is not dependent on DNA or RNA. Co-immunoprecipitation was performed in nuclear extract pretreated with or without Benzonase, which degrades nucleic acids in the lysate. Proteins were resolved in SDS-PAGE and probed with indicated antibodies. A previously known MeCP2-interacting protein, HDAC1, is shown as a control.
(TIF)

S2 Fig Deletion of amino acids 163–380 in MeCP2 disrupted interaction with LEDGF. (a) Western blot analysis of Myc-LEDGF/p75 in anti-HA Immunoprecipitate. HA-MeCP2 and Myc-LEDGF/p75 were co-transfected into HEK293 cells. Anti-HA immunoprecipitate was resolved in SDS-PAGE and probed with anti-Myc antibody (Top panel). HA-MeCP2 (middle panel) and Myc-LEDGF/p52 (bottom panel) in input were also analyzed by Western blot. Schematics below blots show the configuration of each MeCP2 deletion construct. (b) Western blot analysis of HA-MeCP2 in anit-Myc immunoprecipitate. HA-MeCP2 and Myc-LEDGF/p52 were co-transfected into HEK293 cells. Anti-Myc immunoprecipitate was resolved in SDS-PAGE and probed with anti-HA antibody (Top panel). HA-MeCP2 (middle panel) and Myc-LEDGF/p52 (bottom panel) in input were also analyzed by Western blot. Arrow indicates where MeCP2^Δ163–380^ band would be if there is one. Star indicates the MeCP2^Δ381–492^ band overlapping with IgG. Schematics below blots show the configuration of each MeCP2 deletion construct.
(TIF)

S3 Fig Differential gene expression analysis of RNA-Seq data. (a-b) Quantification of MeCP2-repressed genes (a) and MeCP2-activated genes (b) in WT and *Mecp2* KO cortex by RT-qPCR. Three to four previously known targets and three novel targets identified only in our RNA-Seq data in each group were selected for validation. Mean ± S.E.M is plotted; n = 5–6 per genotype. ** *P* < 0.01, *** *P* < 0.001; two-tailed *t*-test with Benjamini-Hochberg correction. (c) Good correlation between qRT-PCR result and RNA-Seq analysis result of the 13 genes analyzed in a-b. r, correlation coefficient.
(TIF)

S4 Fig Functional enrichment analysis of genes with splicing changes, alignment of MeCP2 ChIP signal on all genes in the genome, and alignment of mCG%, mCH%, and hmC% on all exons in the genome. (a) Significant terms (Benjamini *P*-value < 0.05) were shown with one redundant term omitted. (b) Read counts per millions of mapped reads (ChIP minus input) across regions spanning from 2,000bp upstream and 2,000bp downstream of all genes. Coverage for ChIP and input data are calculated for each biological replicate and normalized to be equal length across all the mm9 genes by sampling at equal intervals. Displayed is the average ChIP minus input profile of two biological replicates. TSS: transcription start site. TES: transcription end site.(c) Percentage of CG methylation across regions spanning from 500bp upstream and 500bp downstream of all exons in the genome. (d) Percentage of CH methylation across regions spanning from 500bp upstream and 500bp downstream of all exons in the genome. (e) Percentage of hydroxymethylation across regions spanning from 500bp upstream and 500bp downstream of all exons in the genome.
(TIF)

S5 Fig Validation of splicing changes by qRT-PCR. For each splicing event, specific primers for inclusion isoform and exclusion isoform were designed. Inc / Exc ratio was calculated using 2^-ΔCt^ method. Mean ± S.E.M is plotted; n = 5–6 per genotype. * *P* < 0.05, ** *P* < 0.01, *** *P* < 0.001; two-tailed *t*-test with Benjamini-Hochberg correction.
(TIF)

S6 Fig Raw data tracks of MeCP2 ChIP-seq reads and input reads over the entire *Gria2* gene locus. (TIF)

S7 Fig Validation of MeCP2 peaks identified by ChIP-Seq. ChIP-qPCR was performed using primers specific to each peak. Mean ± S.E.M; n = 4; * *P* < 0.05, ** *P* < 0.01, *** *P* < 0.001; one-way ANOVA followed with Holm-Sidak's multiple comparisons test.
(TIF)

S8 Fig Gria2 flip/flop splicing is altered in multiple brain regions of *Mecp2* KO mouse and the hypothalamus of *Mecp2* overexpression (OE) mouse. RNA-Seq read density around flip and flop exon in indicated brain region of WT and KO (or OE) mouse. Percentage of flip isoform (φ) is shown to the right of density plot. Δφ (KO or OE—WT) was calculated and the Bayes factor (BF) is shown below. Difference in Δφ among different studies might reflect difference of brain region, the knockout allele (cortex data was generated from the Jaenisch allele and the others Bird allele), age of mice when tissue was collected (cortex: 6 weeks of age; hypothalamus: 7 weeks of age; visual cortex: 8–9 weeks of age) and other experimental conditions.
(TIF)

S9 Fig Engineering splice factor (ESF) can modulate *Gria2* flip/flop splicing. (a) Schematic diagram of the *Gria2* minigene. The ESF binding site on flip or flop exon is shown. (b-c) Quantification of *Gria2* flip/flop ratio (b) and total *Gria2* mRNA level (c) in primary culture neurons infected with AAV-Ctrl or AAV-ESF-flop-Gly. Mean ± S.E.M of three independent experiments, ** *P* < 0.01; two-tailed *t*-test.
(TIF)

S1 Table MeCP2-interacting proteins identified by coIP-MS. (XLSX)

S2 Table Gene expression fold change (log_2_FC[KO/WT]) of 315 MeCP2-repressed genes defined by a recent meta-analysis. (XLSX)

S3 Table Lists of splicing events significantly changed in *Mecp2* KO cortex. (XLSX)

S4 Table Summary of MISO analysis comparisons between our study, Chen et al 2015 PNAS and Gabel et al 2015 Nature. (XLSX)

S5 Table Lists of overlapping splicing changes between our study (cortex) and Chen et al, 2015, PNAS (hypothalamus). (XLSX)

S6 Table Lists of overlapping splicing changes between our study (cortex) and Gabel et al 2015 Nature [9] (Visual cortex). (XLSX)

S7 Table Lists of MeCP2 ChIP-Seq peaks (Highlighted peaks were validated by ChIP-qPCR.). (XLSX)

S8 Table List of all PCR primers used in our study. (XLSX)
