# Supplementary figures and images for: Misregulation of Alternative Splicing in a Mouse Model of Rett Syndrome
Source: PLoS Genet. 2016 Jun 28;12(6):e1006129. doi: 10.1371/journal.pgen.1006129 (PMC4924826; doi:10.1371/journal.pgen.1006129)

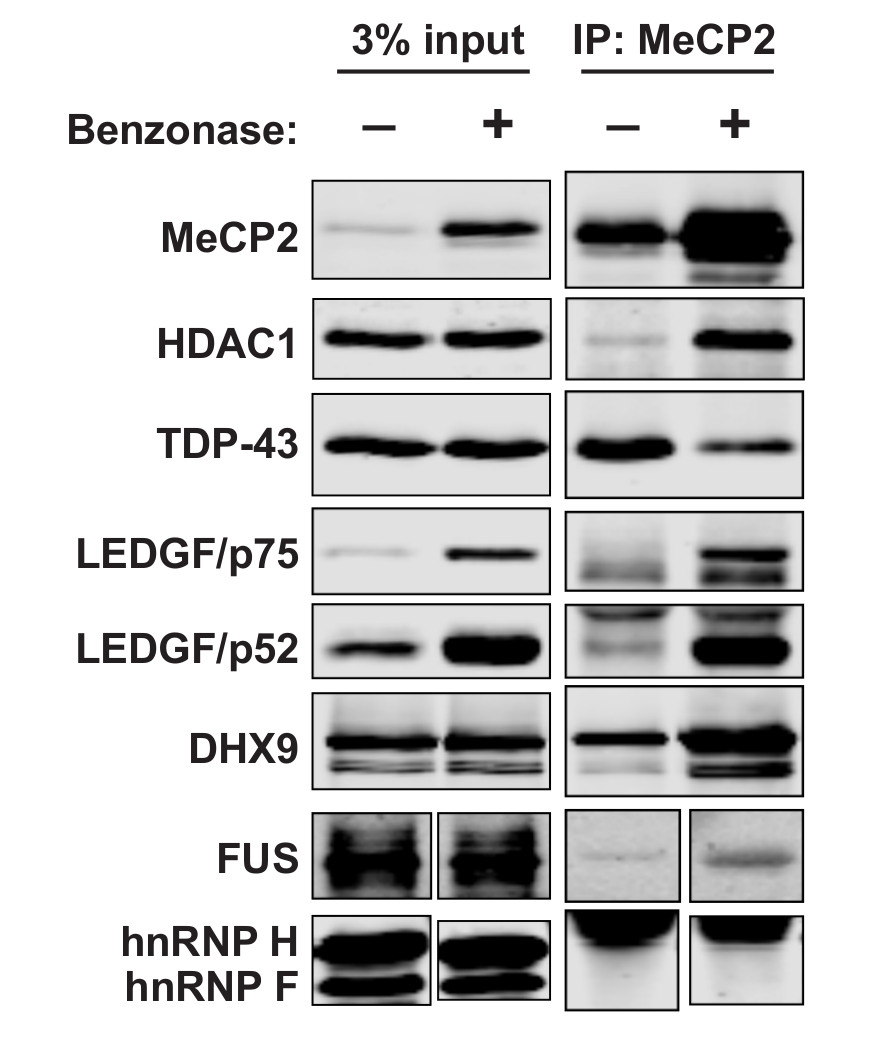

Supplement: S1 Fig — Co-immunoprecipitation was performed in nuclear extract pretreated with or without Benzonase, which degrades nucleic acids in the lysate. Proteins were resolved in SDS-PAGE and probed with indicated antibodies. A previously known MeCP2-interacting protein, HDAC1, is shown as a control. (TIF) [file pgen.1006129.s001.tif]

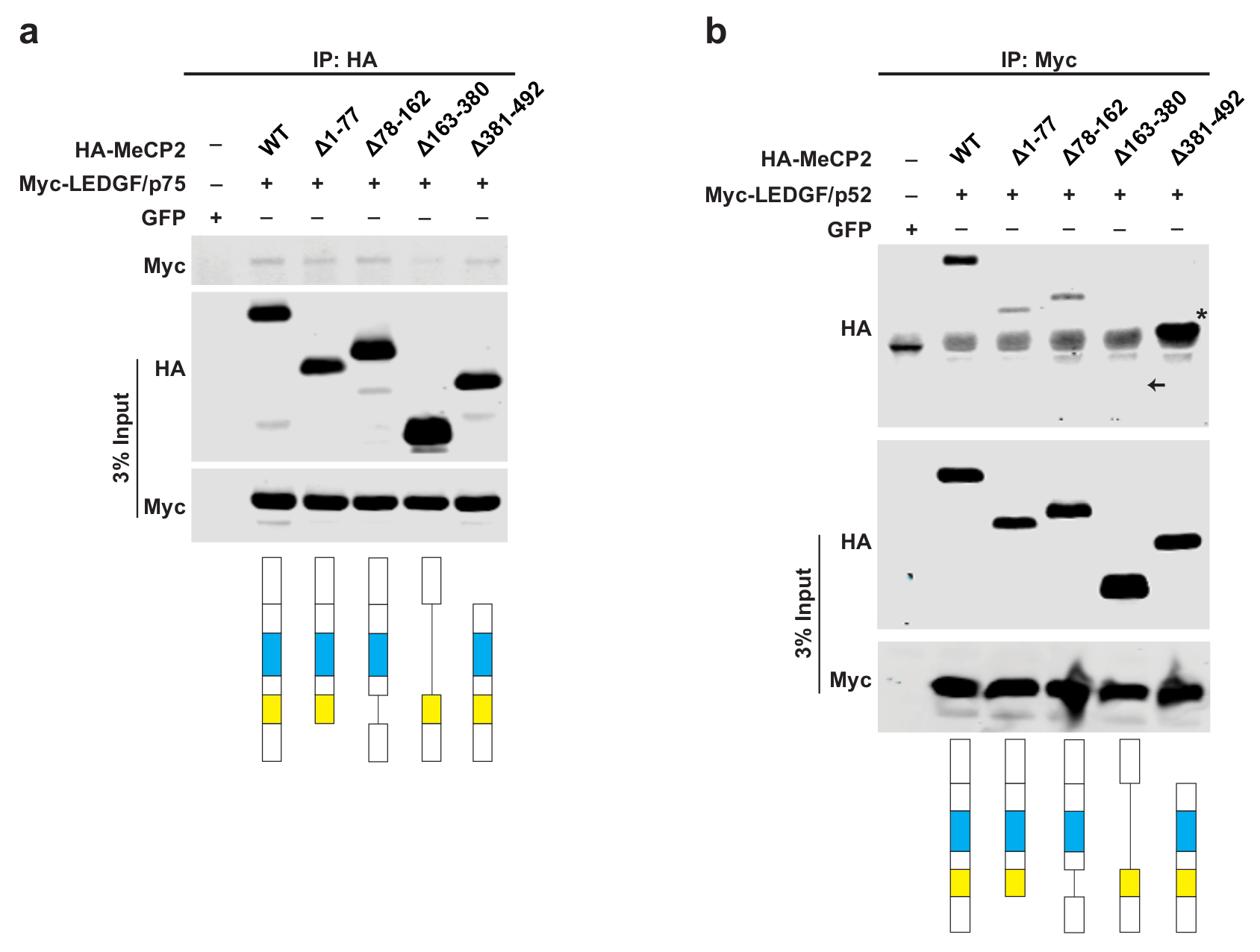

Supplement: S2 Fig — (a) Western blot analysis of Myc-LEDGF/p75 in anti-HA Immunoprecipitate. HA-MeCP2 and Myc-LEDGF/p75 were co-transfected into HEK293 cells. Anti-HA immunoprecipitate was resolved in SDS-PAGE and probed with anti-Myc antibody (Top panel). HA-MeCP2 (middle panel) and Myc-LEDGF/p52 (bottom panel) in input were also analyzed by Western blot. Schematics below blots show the configuration of each MeCP2 deletion construct. (b) Western blot analysis of HA-MeCP2 in anit-Myc immunoprecipitate. HA-MeCP2 and Myc-LEDGF/p52 were co-transfected into HEK293 cells. Anti-Myc immunoprecipitate was resolved in SDS-PAGE and probed with anti-HA antibody (Top panel). HA-MeCP2 (middle panel) and Myc-LEDGF/p52 (bottom panel) in input were also analyzed by Western blot. Arrow indicates where MeCP2Δ163–380 band would be if there is one. Star indicates the MeCP2Δ381–492 band overlapping with IgG. Schematics below blots show the configuration of each MeCP2 deletion construct. (TIF) [file pgen.1006129.s002.tif]

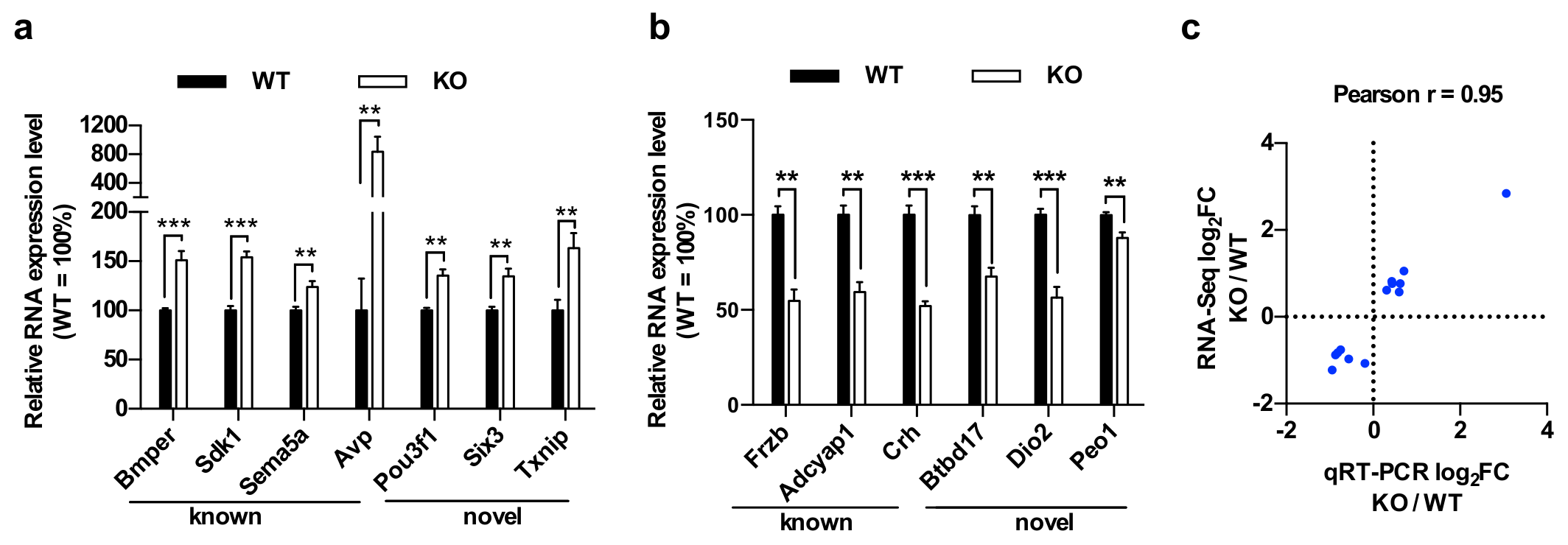

Supplement: S3 Fig — (a-b) Quantification of MeCP2-repressed genes (a) and MeCP2-activated genes (b) in WT and Mecp2 KO cortex by RT-qPCR. Three to four previously known targets and three novel targets identified only in our RNA-Seq data in each group were selected for validation. Mean ± S.E.M is plotted; n = 5–6 per genotype. ** P < 0.01, *** P < 0.001; two-tailed t-test with Benjamini-Hochberg correction. (c) Good correlation between qRT-PCR result and RNA-Seq analysis result of the 13 genes analyzed in a-b. r, correlation coefficient. (TIF) [file pgen.1006129.s003.tif]

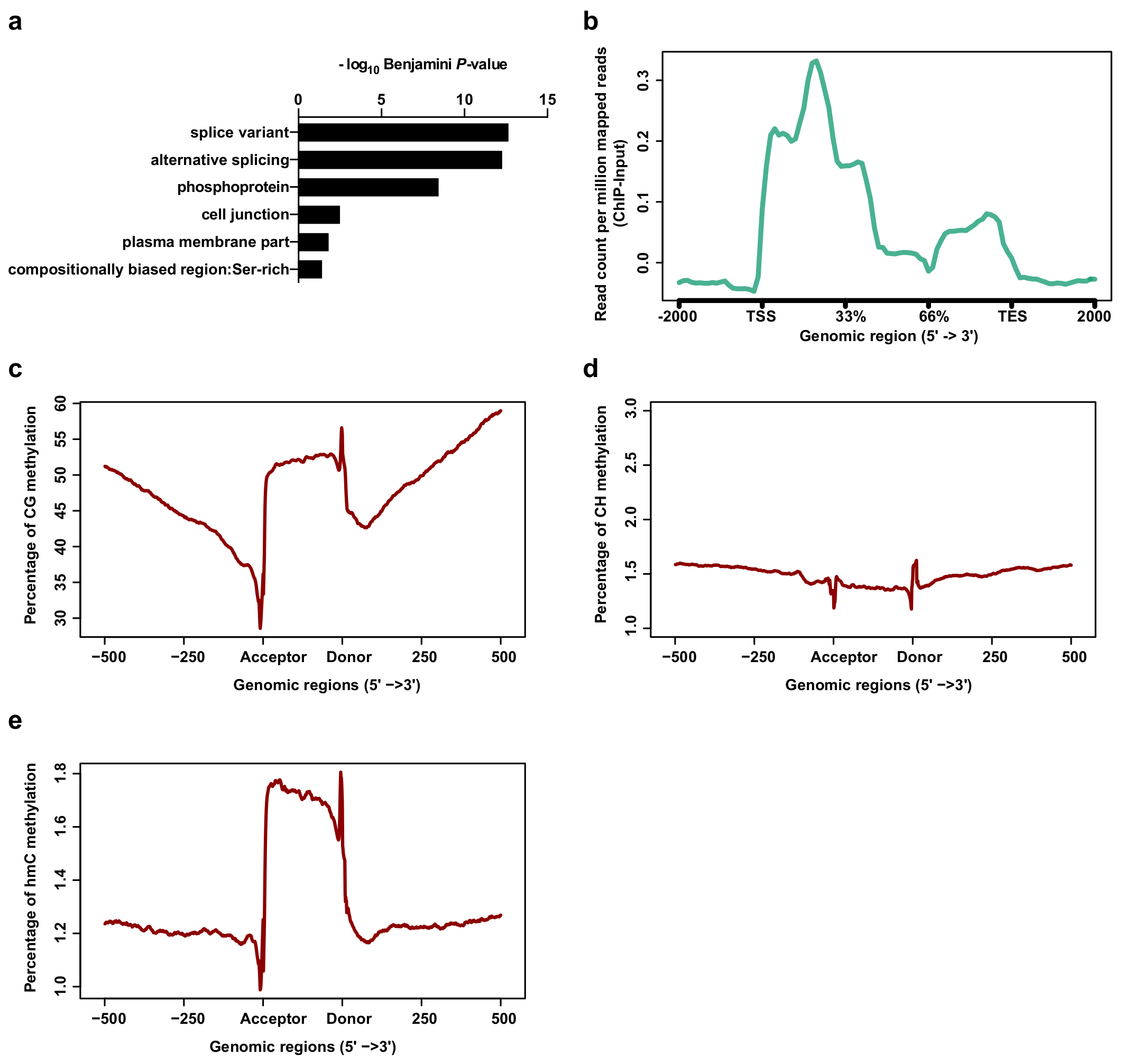

Supplement: S4 Fig — (a) Significant terms (Benjamini P-value < 0.05) were shown with one redundant term omitted. (b) Read counts per millions of mapped reads (ChIP minus input) across regions spanning from 2,000bp upstream and 2,000bp downstream of all genes. Coverage for ChIP and input data are calculated for each biological replicate and normalized to be equal length across all the mm9 genes by sampling at equal intervals. Displayed is the average ChIP minus input profile of two biological replicates. TSS: transcription start site. TES: transcription end site.(c) Percentage of CG methylation across regions spanning from 500bp upstream and 500bp downstream of all exons in the genome. (d) Percentage of CH methylation across regions spanning from 500bp upstream and 500bp downstream of all exons in the genome. (e) Percentage of hydroxymethylation across regions spanning from 500bp upstream and 500bp downstream of all exons in the genome. (TIF) [file pgen.1006129.s004.tif]

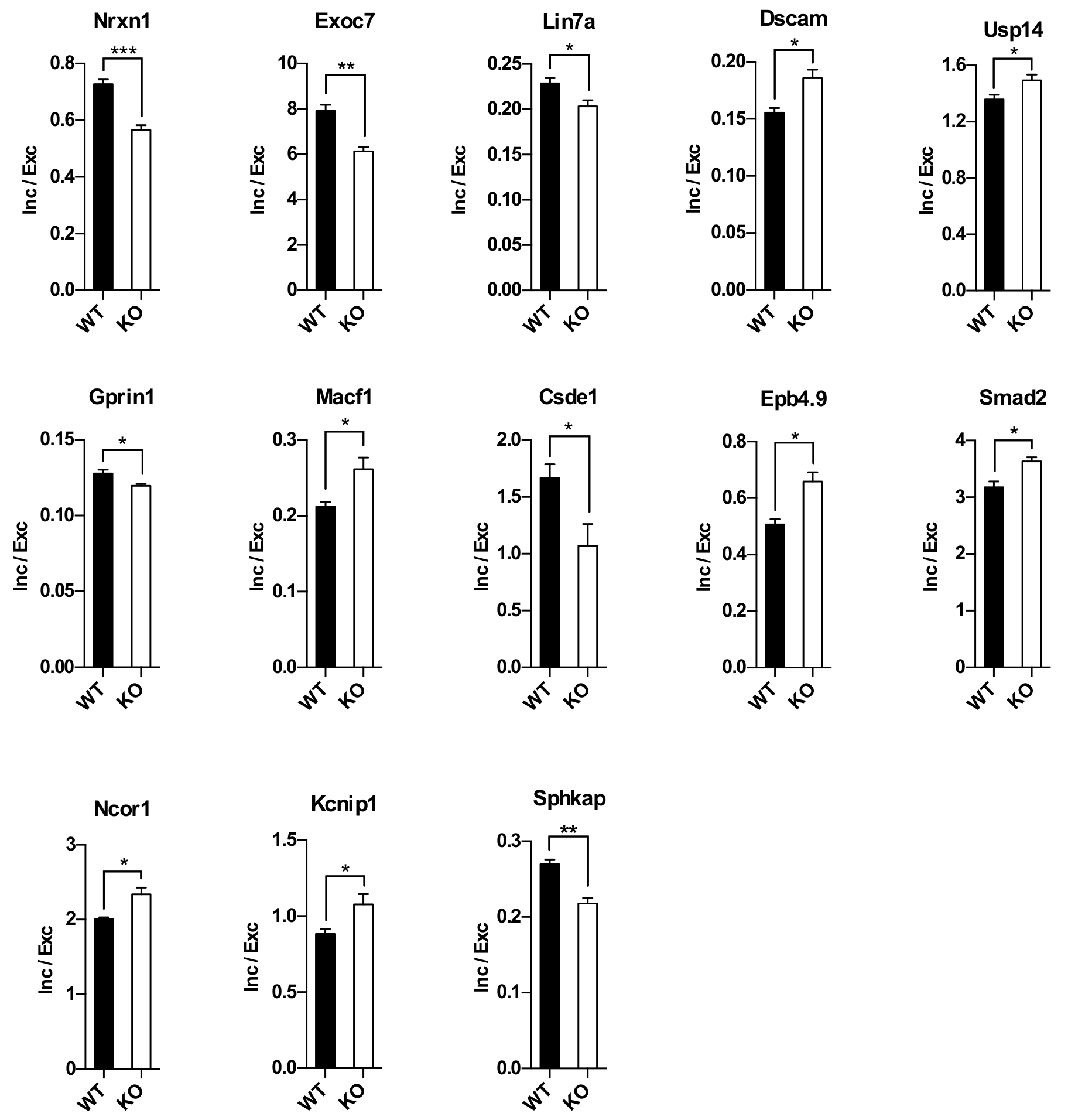

Supplement: S5 Fig — For each splicing event, specific primers for inclusion isoform and exclusion isoform were designed. Inc / Exc ratio was calculated using 2-ΔCt method. Mean ± S.E.M is plotted; n = 5–6 per genotype. * P < 0.05, ** P < 0.01, *** P < 0.001; two-tailed t-test with Benjamini-Hochberg correction. (TIF) [file pgen.1006129.s005.tif]

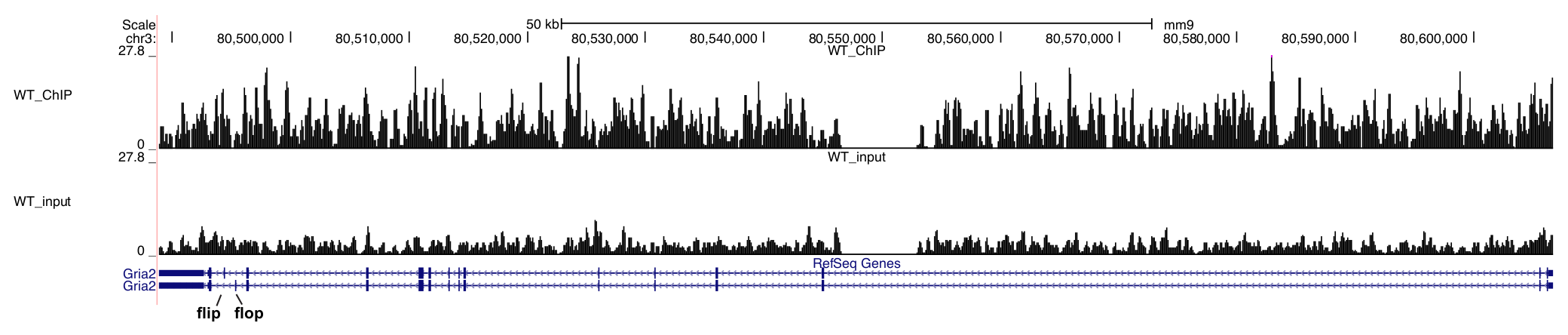

Supplement: S6 Fig — (TIF) [file pgen.1006129.s006.tif]

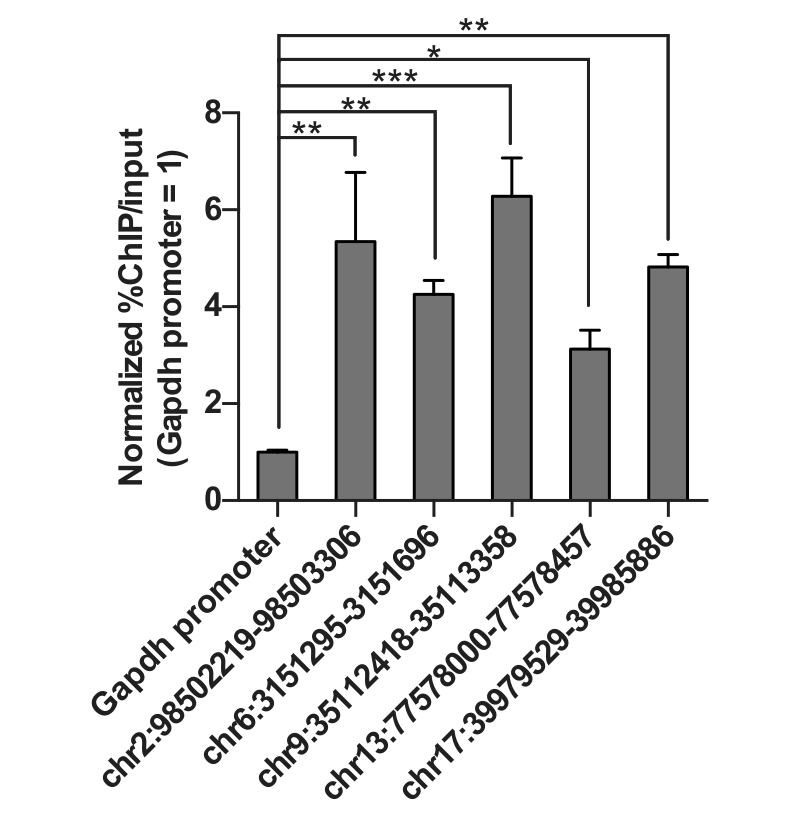

Supplement: S7 Fig — ChIP-qPCR was performed using primers specific to each peak. Mean ± S.E.M; n = 4; * P < 0.05, ** P < 0.01, *** P < 0.001; one-way ANOVA followed with Holm-Sidak's multiple comparisons test. (TIF) [file pgen.1006129.s007.tif]

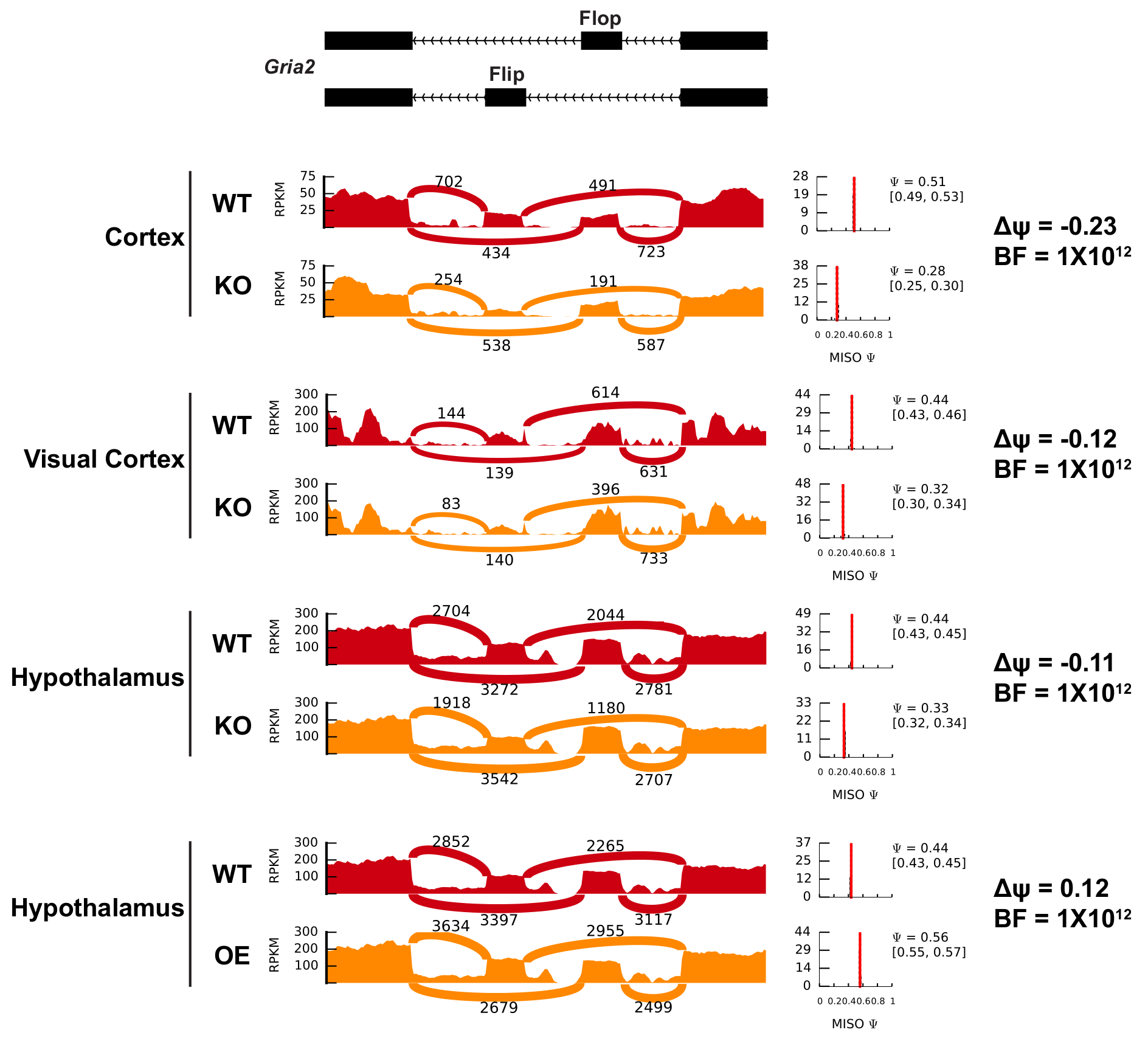

Supplement: S8 Fig — RNA-Seq read density around flip and flop exon in indicated brain region of WT and KO (or OE) mouse. Percentage of flip isoform (φ) is shown to the right of density plot. Δφ (KO or OE—WT) was calculated and the Bayes factor (BF) is shown below. Difference in Δφ among different studies might reflect difference of brain region, the knockout allele (cortex data was generated from the Jaenisch allele and the others Bird allele), age of mice when tissue was collected (cortex: 6 weeks of age; hypothalamus: 7 weeks of age; visual cortex: 8–9 weeks of age) and other experimental conditions. (TIF) [file pgen.1006129.s008.tif]

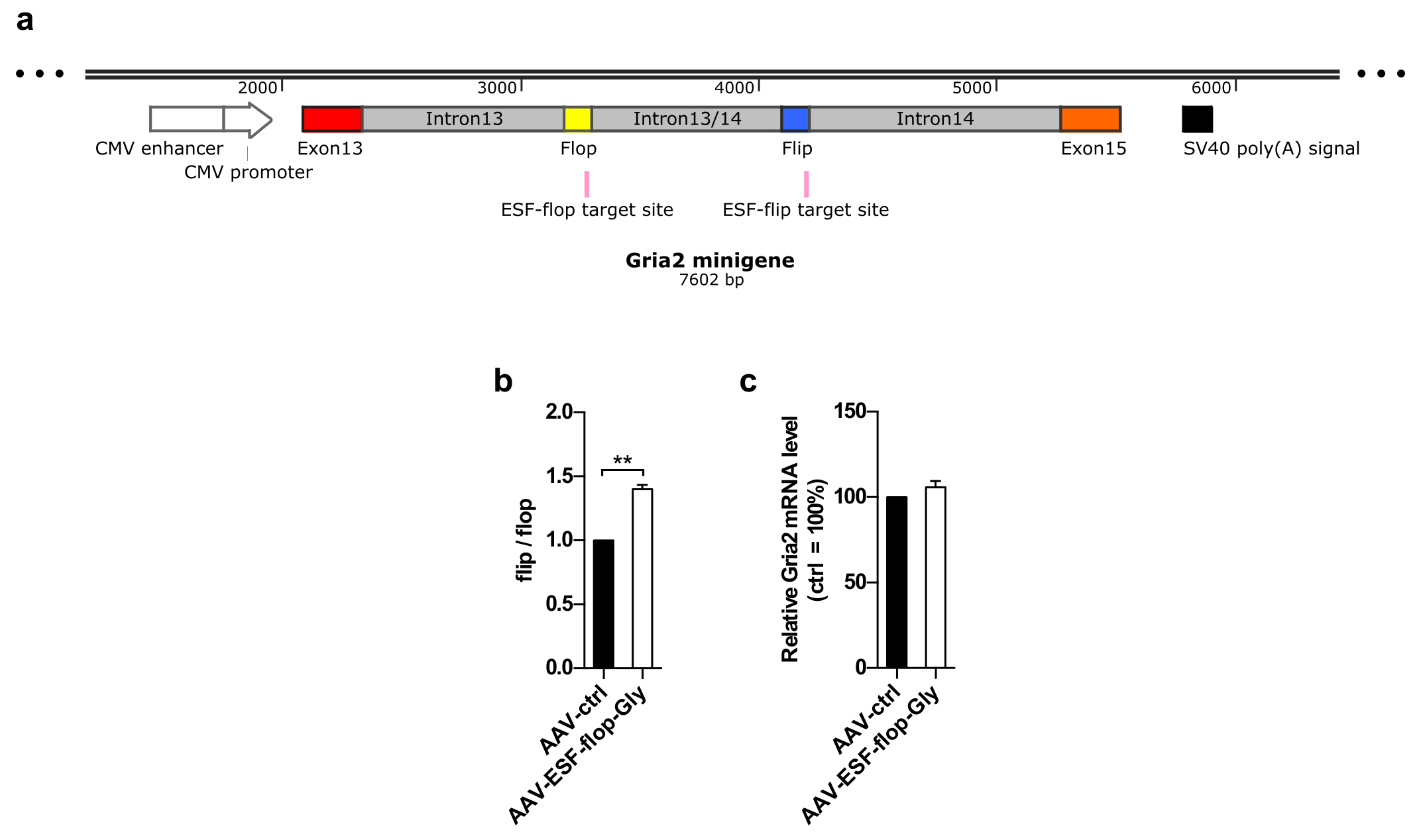

Supplement: S9 Fig — (a) Schematic diagram of the Gria2 minigene. The ESF binding site on flip or flop exon is shown. (b-c) Quantification of Gria2 flip/flop ratio (b) and total Gria2 mRNA level (c) in primary culture neurons infected with AAV-Ctrl or AAV-ESF-flop-Gly. Mean ± S.E.M of three independent experiments, ** P < 0.01; two-tailed t-test. (TIF) [file pgen.1006129.s009.tif]
